# Effects of sodium nitroprusside and salicylic acid applications on morphological, physiological and biochemical properties of Garnem (*Prunus dulcis* × *Prunus persica*) rootstock against alkaline stress under in vitro conditions

**DOI:** 10.1186/s12870-026-08300-8

**Published:** 2026-02-18

**Authors:** Necla Şaşkın, Bekir Erol Ak, Heydem Eki̇nci̇, Ali Sarioğlu

**Affiliations:** 1 Department of Horticulture, Faculty of Agriculture, Dicle University, Diyarbakır, 21280 Türkiye; 2 Department of Horticulture, Faculty of Agriculture, Harran University, Şanlıurfa, 63050 Türkiye; 3 Department of Soil Science, Faculty of Agriculture, Harran University, Şanlıurfa, 63050 Türkiye

**Keywords:** Alkaline stress, Chlorophyll content, Garnem rootstock (*Prunus dulcis* × *Prunus persica*), In vitro rooting, Oxidative stress, Salicylic acid, Sodium nitroprusside, Stress-alleviating treatments

## Abstract

Alkaline stress is considered as one of the major abiotic stress factors limiting plant production on a global scale. This study was conducted in vitro to evaluate the physiological, morphological, and biochemical responses of Garnem rootstock to NaHCO_3_-induced alkaline stress using sodium nitroprusside (SNP) and salicylic acid (SA) treatments. The aim was to alleviate the negative effects of alkaline stress. The study was conducted during the rooting stage of Garnem rootstock plantlets obtained through in vitro micropropagation. SNP and SA (50, 100, and 150 µM) were applied in vitro to counteract alkaline stress induced by NaHCO_3_ (0 mM, 20 mM, and 40 mM) at various concentrations. As the severity of alkaline stress increased, damage occurred to morphological, physiological, and biochemical parameters. The SNP and SA treatments alleviated the harmful effects of alkaline stress. In the study, the highest survival rate was observed with 0 mM NaHCO_3_ + 50 µM SA (98.33%) and 50 µM SNP (95.00%), while the lowest survival rate was observed with 40 mM NaHCO_3_ + 150 µM SA (6.67%). The longest shoots were observed with 0 mM NaHCO_3_ + 100 µM SNP (4.25 cm), and the shortest shoots were observed with 40 mM NaHCO_3_ + 150 µM SA (0.77 cm). The highest number of leaves was found with 20 mM NaHCO_3_ + 50 µM SA (20.63 per plantlet), and the lowest number of leaves was found with 20 mM NaHCO_3_ + 150 µM SNP (5.33 per plantlet). The highest plant fresh weight was observed with the 0 mM NaHCO_3_ + 50 µM SNP (2.07 g), while the lowest plant fresh weight was observed with the 20 mM NaHCO_3_ + 150 µM SA (0.14 g) application. The highest plant dry weight was observed with the 0 mM NaHCO_3_ + 150 µM SA (0.24 g), while the lowest plant dry weight was observed with the 20 mM NaHCO_3_ + 150 µM SA and 40 mM NaHCO_3_ + 150 µM SA (0.04 g) applications. The highest injury was observed with 40 mM NaHCO_3_+ 50 µM SA, 40 mM NaHCO_3_+ 100 µM SA and 40 mM NaHCO_3_+ 150 µM SA (4.00) and the lowest injury was observed with 0 mM NaHCO_3_+50 µM SNP, 0 mM NaHCO_3_+100 µM SNP and 0 mM NaHCO_3_+150 µM SNP (1.00). Membrane permeability increased under high alkaline stress, while 100 µM SNP applications reduced this rate, contributing to the preservation of cellular structure. All SNP and 50 µM SA applications increased leaf relative water content in response to alkaline stress. Significant decreases in chlorophyll a, chlorophyll b, and total chlorophyll concentrations were observed under alkaline stress conditions. However, SNP treatments alleviated these negative effects and increased chlorophyll levels. Furthermore, SNP treatments significantly reduced hydrogen peroxide (H_2_O_2_) and malondialdehyde (MDA) accumulations. Overall, the results demonstrated that SNP treatments at all tested concentrations effectively mitigated NaHCO_3_-induced alkaline stress by improving morphological traits, maintaining membrane integrity, enhancing leaf relative water content and reducing oxidative damage in Garnem rootstock plantlets. In contrast, salicylic acid exhibited a concentration-dependent response, with low doses (50–100 µM) alleviating stress effects, whereas the high concentration (150 µM) induced phytotoxicity and markedly suppressed plant growth and physiological performance.

**Supplementary Information:**

The online version contains supplementary material available at 10.1186/s12870-026-08300-8.

## Introduction

The most critical decision in modern orchard establishments is the selection of rootstock. The soil characteristics and climate conditions of the region where the garden will be established are the main criteria taken into consideration in rootstock selection. Rootstock has a direct impact on the plant’s growth and development parameters. Rootstock is an important factor in increasing the yield potential of the plant, its resistance to environmental stresses and product quality. It ensures harmonious plant development in regions with diverse soil types. It provides resistance to soil-borne diseases and pests. The variety grafted onto the rootstock increases the yield potential of the plant and the product quality [1, 2].

Garnem (*Prunus dulcis* × *Prunus persica*), an important rootstock for the cultivation of *Prunus* species, is a vigorous rootstock and grows very well in nurseries. It is resistant to iron chlorosis. It adapts successfully to irrigated and well-drained low-fertility soils and has a high tolerance to arid conditions. Garnem rootstock has lower resistance to excessive lime than GF-677 rootstock. Garnem rootstock provides full graft compatibility with all almond, peach, and nectarine varieties. Furthermore, this rootstock propagates very well in vitro [3–7].

Micropropagation, a plant tissue culture method, is preferred over other vegetative propagation methods because it allows for the production of a large number of uniformly structured plants in a small area in a short time, preserves plant genetics, provides plant material free of diseases and pests, enables year-round production regardless of the growing season, and enables the propagation and protection of endangered plants [8–11].Screening stress tolerance using tissue culture methods under in vitro conditions is a highly effective way to elucidate changes in physiological and biochemical properties under stressful conditions and to improve stress tolerance. The vast majority of stress studies are conducted in field conditions. However, conducting studies in this way is physically and economically challenging, and various risks due to external conditions can harm the study. Therefore, stress studies in vitro are conducted in a more limited area and under controlled conditions. This is a more effective and economical method compared to studies conducted in field conditions [12]. Soil salinity and alkalinity due to global climate change pose serious problems for plant growth in agricultural production. Under salinity stress conditions, high amounts of dissolved salts accumulate on the soil surface or in the lower layers. Soil stress is divided into three categories based on salt content and pH. These are classified as mild (salt content < 3‰, pH = 7.1–8.5), moderate (salt content = 3–6‰, pH = 8.5–9.5), and severe (salt content > 6‰, pH > 9.5 [13]. Salt stress is caused by NaCl, Na_2_SO_4_, and other neutral salts. Under these conditions, sodium ions directly enter the cell through channel and transport proteins, causing ion toxicity in the plant. High ion concentrations outside the cell reduce the osmotic potential, drawing water molecules out of the cell. In this situation, water uptake by plants becomes difficult and causes physiological drought in the plant. This condition inhibits plant growth. Alkaline stress is caused by NaHCO_3_ and Na_2_CO_3_. In addition to salt stress, these substances also increase the soil pH. Alkaline stress is defined as a soil pH above 7.5. The symptoms of this stress on plants include wilting and yellowing of dehydrated leaves. Under these stress conditions, in addition to salt stress, plants are exposed to high pH, ​​resulting in ion toxicity and osmotic stress. High pH levels significantly disrupt intracellular pH balance, negatively affecting cell membrane integrity and causing cellular damage. Severe damage to plant growth and development, as well as to physiological and biochemical metabolism, occurs in saline-alkaline conditions. In addition to reducing plant root vigor, it also reduces photosynthesis rate, stomatal conductance, transpiration rate, and photosynthetic pigments. At high pH levels, due to the antagonistic effect between plant nutrients, nutrient uptake becomes more difficult, negatively affecting biomass, crop yield, and quality [14–19].

Various stress-alleviating agents are used to improve plant performance under abiotic and biotic stress conditions. These agents alleviate the negative effects on the growth and development of stressed plants, resulting in better plant growth. Of these stress-alleviating agents, sodium nitroprusside (SNP) and salicylic acid (SA) promote plant survival and growth under abiotic stress conditions. It alleviates the effects of oxidative damage by activating the plant’s antioxidant defense mechanism [20, 21].

Sodium nitroprusside (SNP) is a nitric oxide donor with the chemical formula Na_2_[Fe(CN)_5_NO]·2H_2_O. Nitric oxide (NO) is a small, gaseous free radical with a half-life of less than six seconds. It plays an important role in many physiological and biochemical processes in plants. NO and its donors are widely used to enhance plant responses to stress conditions, as well as growth and development. Sodium nitroprusside is the most commonly used NO donor molecule. SNP increases plant tolerance to stress by reducing oxidative damage under environmental stress conditions. SNP also protects plants from the harmful effects of oxidative stress by increasing antioxidant enzyme activity. It has positive effects on seed germination, root growth, leaf expansion, and plant development. It improves photosynthesis in plants. It delays leaf fall and senescence. Sodium nitroprusside is widely used in plant tissue culture protocols. SNP added to plant tissue culture media not only increases shoot and root formation but also increases micropropagation success. It positively affects plant growth and development when used alone or in combination with plant growth regulators. Using high doses of SNP can have harmful and toxic effects [22–25].

Salicylic acid (SA) is a naturally occurring phenolic compound, 2-hydroxybenzoic acid. It has a colorless crystalline structure. The word “salicylic” is derived from *Salix*, the latin name for the willow tree. Salicylin, a glycoside of salicylic acid, was first isolated from willow bark in 1826. Found in all plant groups, salicylic acid is classified as a plant growth regulator [26, 27]. It has effects on plant physiological and biochemical processes. It affects important enzyme activities, ion uptake, growth, and development in plants. In addition to having positive effects on flowering, it can reverse the effects of abscisic acid on leaf fall. Influencing photosynthetic rates and thermogenesis are also among the primary functions of salicylic acid [28]. It also promotes seed germination and root development. It plays a direct role in ameliorating the negative effects of abiotic stress factors such as drought, salinity, heavy metals, etc [7, 29–35].

In this study, it is assumed that alkaline stress induced by NaHCO_3_ application under in vitro conditions in Garnem (*Prunus dulcis* × *Prunus persica*) rootstock causes negative changes in the morphological, physiological, and biochemical characteristics of the plants; and that sodium nitroprusside (SNP) and salicylic acid (SA) applications may affect the responses induced by this stress in the plant as well as the level of stress symptoms. In addition, it is considered that the application of SNP and SA at different concentrations may result in different responses in parameters such as survival rate under alkaline stress, root and shoot development, number of leaves, chlorophyll content, membrane permeability, and indicators of oxidative stress.

## Material and method

### Plant material

The study was conducted in the Plant Tissue Culture Laboratory of the Department of Horticulture, Faculty of Agriculture, Harran University, where the rootstock Garnem (*Prunus dulcis × Prunus persica*), a hybrid of peach and almond, was used as plant material. The rootstock, true to its name, was supplied by BETA Fidancılık (Adana, Turkey).

### Sterilization and shoot initiation stage

**Fig. 1:**
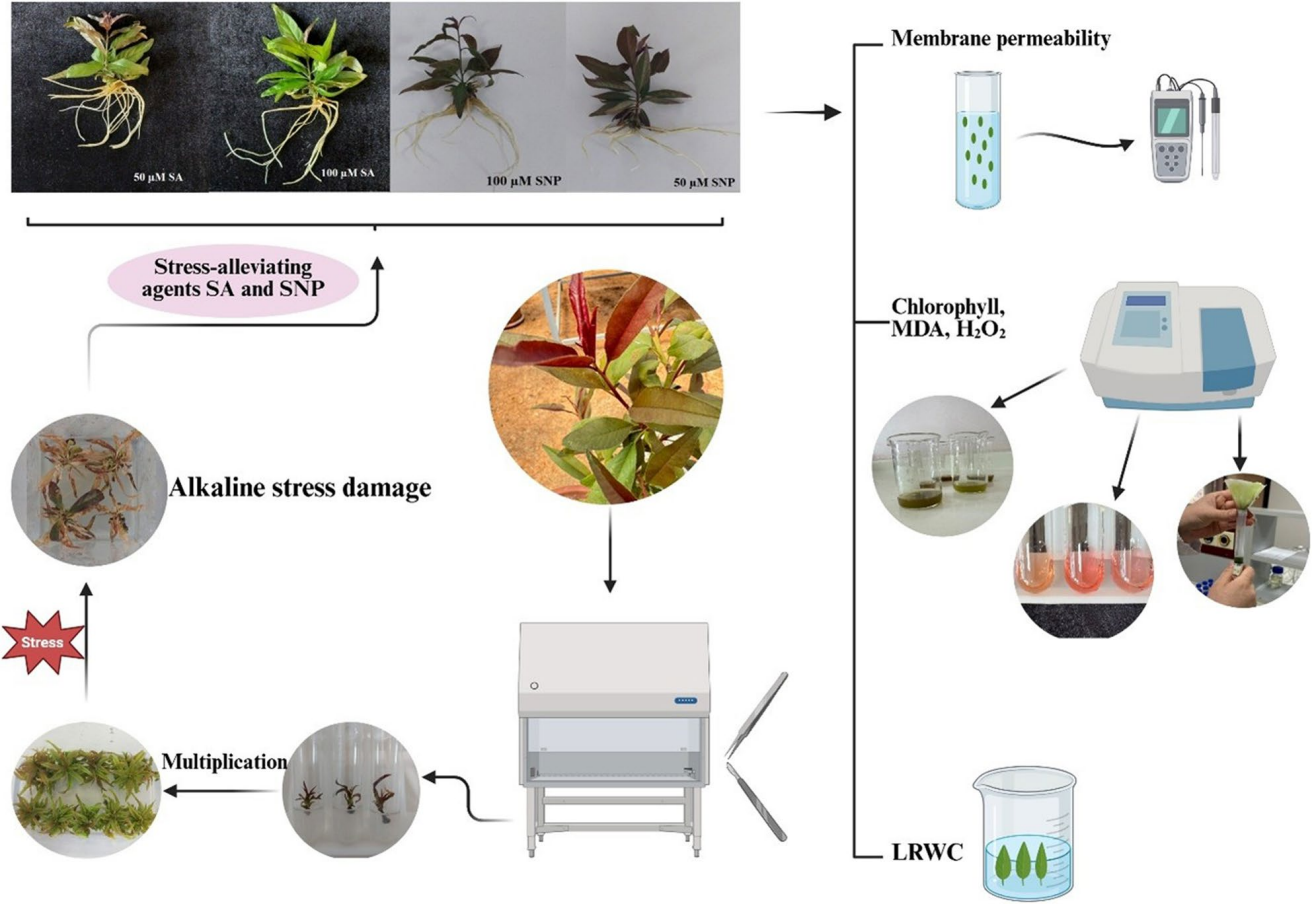
Flow chart of the experimental study showing the effects of salicylic acid (SA) and sodium nitroprusside (SNP) applications on the morphological, physiological and biochemical parameters of plantlets under alkaline stress conditions (*LRWC: Leaf Relative Water Content, MDA: Malondialdehyde, H_2_O_2_: Hydrogen peroxide)

Shoots were taken from one-year-old Garnem rootstock during their active growth period, with 3–5 buds on them. The leaves on these shoots were removed and divided into micro cuttings with a single bud. The micro cuttings were subjected to a pre-sterilization process. This process involved soaking the micro cuttings in detergent water for 10 min and then rinsing them with tap water until they were completely clean. Following the completion of pre-sterilization, the micro cuttings were transferred into a sterile cabinet, treated with 70% ethanol for 2 min, and subsequently agitated in a 10% sodium hypochlorite (NaOCl) solution for 12 min. After this treatment, the cuttings were rinsed three times with sterile distilled water. The micro cuttings, following completion of the sterilization process, were prepared for transfer to the shoot proliferation medium [7, 36, 37]. After surface sterilization, the micro cuttings were transferred to Murashige and Skoog (MS) medium containing 30 g L^− 1^ sucrose, 2.0 mg L^− 1^ BAP, 0.4 mg L^− 1^ GA_3_, and 5.5 g L^− 1^ agar as the shoot proliferation medium. To prevent bacterial contamination in the culture media, 1.2 ml L^− 1^ PPM (Plant Preservative Mixture) was added to the media [38–40]. The pH of the culture media was adjusted to 5.8. Plants transferred to the culture medium were maintained in a growth chamber under 65 µmol m^− 2^ s^− 1^ white fluorescent lighting, a 16:8 h photoperiod, 65–70% relative humidity, and a temperature of 25 ± 1 °C for 4 weeks (Fig. 1).

### Shoot multiplication stage

Following the transfer of micro cuttings, after approximately 3–4 weeks, with the initiation of shoot formation, the plantlets were transferred to the multiplication medium to obtain a sufficient number of plantlets for the design of the treatments. During the multiplication stage, the plantlets were used in MS medium containing 30 g L^− 1^ sucrose, 2.0 mg L^− 1^ BAP, and 5.5 g L^− 1^ agar. To prevent bacterial contamination in the culture media, 1.2 ml L^− 1^ PPM (Plant Preservative Mixture) was added to the media [38–40]. The pH of the culture media was adjusted to 5.8. Plants transferred to the culture medium were maintained in a growth chamber under 65 µmol m^− 2^ s^− 1^ white fluorescent lighting, a 16:8 h photoperiod, 65–70% relative humidity, and a temperature of 25 ± 1 °C for 4 weeks (Fig. 1).

### Rooting stage: alkaline stress and stress-alleviating treatments

Once a sufficient number of plantlets were obtained, the study began at the rooting stage. Plantlets were transferred to MS medium containing 30 g L^− 1^ sucrose, 1.5 mg L^− 1^ IBA, and 5.5 g L^− 1^ agar as rooting medium. NaHCO_3_ was added to the rooting medium at concentrations of 0 mM, 20 mM and 40 mM to induce alkaline stress in vitro. The stress-alleviating agents SNP and SA were used at concentrations of 50 µM, 100 µM, and 150 µM. The stress-alleviating agents were added to the medium before autoclaving [41–44]. The study consisted of 21 treatments (Table 1). To prevent bacterial contamination in the culture media at all stages, 1.2 ml L^− 1^ PPM (Plant Preservative Mixture) was added to the media [38–40]. The pH of the culture media was adjusted to 5.8. The experiment was conducted according to a completely randomized design with three replicates per treatment. Each replicate consisted of a total of 20 explants distributed into culture vessels to ensure adequate space and nutrient conditions for plant growth. Plants transferred to the culture medium were maintained in a growth chamber under 65 µmol m^− 2^ s^− 1^ white fluorescent lighting, a 16:8 h photoperiod, 65–70% relative humidity, and a temperature of 25 ± 1 °C for 4 weeks (Fig. 1).

**Table 1:** Different concentrations of NaHCO_3_, SNP and SA and application groups

Treatments
0 mM NaHCO_3_
20 mM NaHCO_3_
40 mM NaHCO_3_
0 mM NaHCO_3_ + 50 µM SNP
0 mM NaHCO_3_ + 100 µM SNP
0 mM NaHCO_3_ + 150 µM SNP
20 mM NaHCO_3_ + 50 µM SNP
20 mM NaHCO_3_ + 100 µM SNP
20 mM NaHCO_3_ + 150 µM SNP
40 mM NaHCO_3_ + 50 µM SNP
40 mM NaHCO_3_ + 100 µM SNP
40 mM NaHCO_3_ + 150 µM SNP
0 mM NaHCO_3_ + 50 µM SA
0 mM NaHCO_3_ + 100 µM SA
0 mM NaHCO_3_ + 150 µM SA
20 mM NaHCO_3_ + 50 µM SA
20 mM NaHCO_3_ + 100 µM SA
20 mM NaHCO_3_ + 150 µM SA
40 mM NaHCO_3_ + 50 µM SA
40 mM NaHCO_3_ + 100 µM SA
40 mM NaHCO_3_ + 150 µM SA

### Morphological, physiological, and biochemical measurements

#### Morphological measurements

##### Survival rate and rooting rate

Approximately four weeks after the experiment was terminated, the survival rate was calculated by dividing the number of surviving plants by the number of plants transferred to the treatment and multiplying by 100, and expressed as a percentage (%). Similarly, the rooting rate was expressed as a percentage (%), calculated by dividing the number of plants that successfully rooted in the rooting medium by the number of plants transferred to the treatment and multiplying by 100 [45, 46].

##### Shoot and root length

Shoot and root lengths of the plantlets were measured in centimeters (cm) using a ruler [47].

##### Number of leaves and root

The number of leaves and roots of each plantlet was counted individually and recorded as one per plantlet [7].

##### Plant fresh weight and dry weight

At the end of the experiment, the roots of the plantlets removed from the nutrient medium were thoroughly cleaned and weighed using a precision scale. Their weight was expressed in g. Plantlets with measured fresh weight were kept in a 70 °C oven for 48 h, after which their dry weight was measured in g [48].

##### Injury index

The injury index was determined using a scale of 1–4 based on visible symptoms of alkaline stress caused by NaHCO_3_. The scale is defined as follows:

1. No injury;
2. Necrosis at the leaf tips and edges;
3. Necrosis throughout the leaf;
4. Dead.

Plants were visually evaluated using this scale. Based on the results, the injury index was calculated using the formula below [49, 50].

Injury Index = Σ (injury level x number of plantlets)/Total number of plantlets.

###  Physiological measurements

#### Membrane permeability

Membrane permeability was calculated by Lutts et al. [51]. Three 1 cm^2^ discs were taken from the leaf and rinsed three times in distilled water in a glass tube. Then, 10 ml of distilled water was added to the closed falcon tubes and shaken on a shaker at 25 °C for 24 h. After 24 h, the electrical conductivity (EC) was first measured (C1). These samples were autoclaved at 120 °C for 20 min. After that, the samples were removed from the autoclave, the temperature was allowed to drop to 25 °C, and the EC was measured again (C2). Membrane permeability was calculated using the formula below:

$$\mathrm{Membrane}\:\mathrm{Permeability}=\mathrm{C1/C2}\times100$$

#### Leaf relative water content (LRWC)

To determine leaf relative water content in this study, the fresh weight of the leaves from each treatment was first measured. These leaves were then soaked in distilled water for 4 h, and then their turgor weights were measured. The weighted leaves were then stored in an oven at 65 °C for 48 h, after which their dry weights were measured. Fresh, turgor, and dry weights of the leaf samples were determined, and the relative water content of the leaves was calculated as a percentage (%) according to the formula below [52, 53]:

$$\mathrm{LRWC}\left(\%\right)=\left(\left(\mathrm{FW}-\mathrm{DW}\right)\right)/\left(\mathrm{TW}-\mathrm{DW}\right)\times100$$

FW: Fresh Weight

DW: Dry Weight

TW: Turgor Weight.

### Biochemical measurements

#### Chlorophyll

At the end of the experiment, chlorophyll content was determined according to Arnon [54]. A 0.5 g leaf sample was taken from the leaves of the treatments. The leaf sample was completely homogenized in 5 ml of an 80% acetone: water solution. Then, it was passed through a paper filter and transferred to light-proof tubes. Readings were taken against the 80% acetone control at 663.5 nm for chlorophyll a and 645 nm for chlorophyll b. The results were calculated using the formula below. The results were calculated as mg/L fresh weight and then expressed as mg chlorophyll g^− 1^. Calculation of chlorophyll concentration in acetone solution:

$$\mathrm{Total}\:\mathrm{chlorophyll}\left(\mathrm{mg/L}\right)=20.2\mathrm{A}645+8.02\mathrm{A}663.5$$

$$\mathrm{Chlorophyll}\:\mathrm{a}\:\left(\mathrm{mg/L}\right)=12.7\mathrm{A}663.5 - 2.69\mathrm{A}645$$

$$\mathrm{Chlorophyll}\:\mathrm{b}\left(\mathrm{mg/L}\right)= 22.9\mathrm{A}645 - 4.68\mathrm{A}663.5$$

#### Malondialdehyde (MDA) analysis

In this study, the MDA content in plant leaves was analyzed using the method developed by Weisany et al. [55]. The analysis began with a 0.2 gram sample taken from plant leaves. Leaf samples were homogenized with 10 ml of trichloroacetic acid solution. The homogenized samples were centrifuged at 12,000 g at + 4 °C. After centrifugation, 1 ml of the supernatant was taken and diluted with 20% TCA solution. Then, 1.5 ml of 0.5% thiobarbituric acid solution was added to this mixture. The prepared samples were rapidly cooled on ice. The samples ready for reading were read using a spectrophotometer at wavelengths of 532 nm and 600 nm. Using the absorbance values ​​obtained from the sample measurements, the amount of MDA was calculated using the following formula:

$$\mathrm{MDA}\:\mathrm{content}=\left[\left(\mathrm{A}532 - \mathrm{A}600\right)/155000\right]\times106$$

#### Hydrogen peroxide (H_2_O_2_) analysis

The method developed by Loreto and Velikova [56] was used to determine the amount of H_2_O_2_ in plant leaves. 0.5 g of leaf sample was taken for H_2_O_2_ measurement. Leaf samples were homogenized using 5 ml of trichloroacetic acid solution. 0.75 ml of the homogenized liquid was taken and set aside for analysis. 0.75 ml of 10 mM K-phosphate buffer (pH 7.0) solution and 0.75 ml of KI (potassium iodide) solution were added to the separated sample liquid. The prepared mixture was read using a spectrophotometer at a wavelength of 390 nm. Hydrogen peroxide has a specific absorbance value at this wavelength. A standard graph was created to determine the hydrogen peroxide content, and the hydrogen peroxide content was calculated based on the absorbance values ​​of the samples. This determined the amount of hydrogen peroxide in the leaf samples.

#### Statistical analysis

The data obtained from the studies were statistically analyzed using the analysis of variance technique in the JMP Pro 13 statistical program. Statistical differences between mean values were determined using the TUKEY test (*p* ≤ 0.05). Hierarchical clustering analysis (HCA) was conducted using R software (Version 4.1.1; R Foundation for Statistical Computing, Vienna, Austria). Network correlation analysis was performed using Python 3 with the Pandas v2.0, NetworkX v3.0, and Matplotlib v3.7 libraries. Python scripts were executed directly in the Linux terminal environment (Ubuntu).

## Results and discussion

### Survival rate (%)

**Fig. 2:**
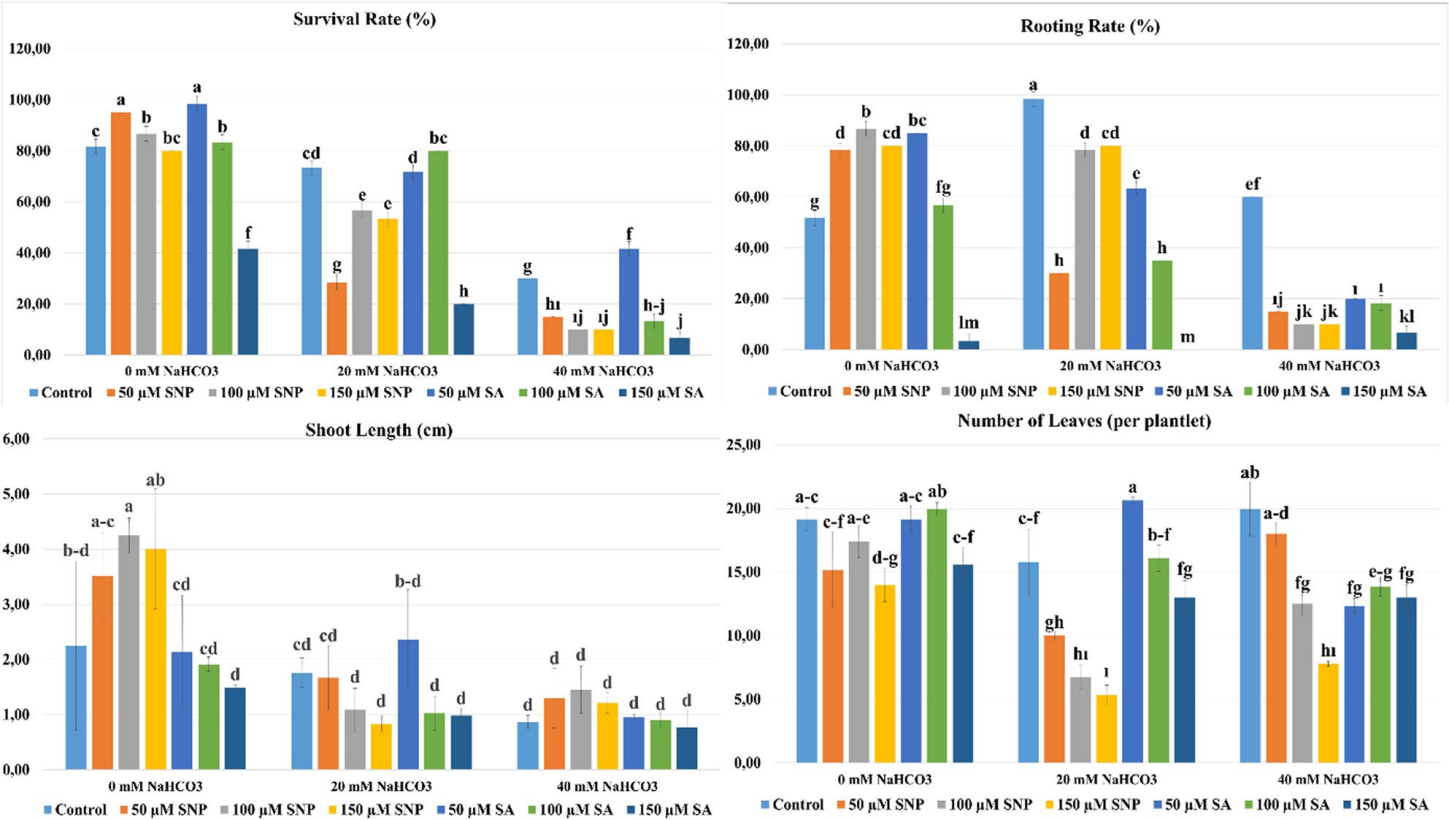
Effect of SNP and SA applications on survival rate, rooting rate, shoot length and number of leaves parameters under NaHCO_3_-induced alkaline stress conditions in vitro (p≤0.05). *The letter groupings used in the statistical analysis indicate differences among the NaHCO_3_× SNP/SA interactions. According to Tukey’s multiple comparison test, mean values denoted by different letters differ significantly at the 5% significance level

The survival results of the study are shown in Fig. 2. The NaHCO_3_ x SNP/SA interaction was found to be statistically significant (*p* ≤ 0.05). The survival rate of plantlets exposed to NaHCO_3_-induced alkaline stress decreased significantly as the NaHCO_3_ concentration increased. At a 0 mM NaHCO_3_ concentration without alkaline stress, the highest survival rate was observed with 50 µM SA (98.33%) and 50 µM SNP (95.00%) applications, while the lowest survival rate was observed with 150 µM SA (41.67%) application. SNP and SA applications increased the survival rate at a 0 mM NaHCO_3_ concentration. In the study, at a NaHCO_3_ concentration of 20 mM, the highest survival rate of the plantlets was observed with the 20 mM NaHCO_3_ + 100 µM SA (80.00%) application, while the lowest survival rate was observed with the 20 mM NaHCO_3_ + 150 µM SA (20.00%) application. As the NaHCO_3_ concentration increased, a very significant decrease occurred, especially at the 40 mM NaHCO_3_ concentration. Application of a low concentration of SA (50 µM SA) improved the survival rate. The highest survival rate was observed with the 40 mM NaHCO_3_ + 50 µM SA (41.67%) application, and the lowest survival rate was observed with the 40 mM NaHCO_3_ + 150 µM SA (6.67%) application. It was determined that the 150 µM SA application had a negative effect on the survival rate at all NaHCO_3_ concentrations. Survival rate is one of the key parameters used to assess the tolerance of plants to biotic and abiotic stress conditions. In this study, a significant decrease in survival rate was observed in parallel with the increase in NaHCO_3_ concentration. This finding can be attributed to NaHCO_3_-induced alkaline stress; ionic toxicity and accompanying osmotic stress, as well as raising the medium pH, severely disrupting cell pH, negatively affecting cell membrane integrity, and consequently restricting plant growth and development [14, 15, 57]. In this study, it was determined that SA application was more effective in increasing the survival rate of plantlets under NaHCO_3_-induced alkaline stress conditions. This finding can be explained by the fact that SA is an important signaling molecule that regulates stress tolerance in plants, mitigating physiological damage caused by abiotic stress by activating antioxidant defense mechanisms at low concentrations, while at high concentrations it can lead to phytotoxic effects [32, 58, 59]. In a study by Sajid and Aftab [60], moderate salicylic acid application to two different potato varieties under salinity stress in vitro increased plant growth, but high concentrations of salicylic acid did not provide tolerance to salinity stress, which is consistent with the results obtained in our study.

### Rooting rate (%)

Rooting rate results are shown in Fig. 2. The NaHCO_3_ x SNP/SA interaction was found to be statistically significant (*p* ≤ 0.05). The highest rooting rate at 0 mM NaHCO_3_ concentration was observed in the 100 µM SNP (86.67%) application, and the lowest rooting rate was observed in the 150 µM SA (3.33%) application. At 20 mM NaHCO_3_ concentration, the highest rooting rate was observed in the 20 mM NaHCO_3_ (98.33%) application, and the lowest rooting rate was observed in the 20 mM NaHCO_3_ + 150 µM SA (0.00%) application. In the study, the highest rooting rate at a 40 mM NaHCO_3_ concentration was observed in the 40 mM NaHCO_3_ (60.00%) application, while the lowest rooting rate was observed in the 40 mM NaHCO_3_ + 150 µM SA (6.67%) application. The study determined that the rooting rate varied significantly depending on both the NaHCO_3_ stress and the applied SA and SNP concentrations. High NaHCO_3_ concentrations are known to inhibit root development by causing high pH and ion toxicity [16, 61]. Under alkaline stress conditions, a significant decrease in ion activity and free concentration of various ions occurs as a result of the precipitation of phosphates and metal ions. Furthermore, under alkaline stress, the metabolism of K^+^ and Na^+^ cations is negatively affected; Na^+^ influx and K^+^ outflow occur, and the intracellular Na^+^/K^+^ ratio increases. Excessive Na^+^ accumulation within the cell can limit the plant’s Ca^2+^ uptake, thus disrupting the integrity of the cell membrane and cell wall structure and hindering signal transduction. Furthermore, the absorption of anions such as Cl^−^, NO_3_^−^, and H_2_PO_4_^−^ is inhibited, resulting in a significant negative charge deficiency and disruption of the metabolic homeostasis of plants [62–65]. In this context, the low rooting rates observed in the study with 40 mM NaHCO_3_ application show that NaHCO_3_ causes oxidative stress in plants and suppresses rooting. The significant decrease in rooting rates at high SA (150 µM) concentrations suggests that SA may increase oxidative levels, creating an additional stress source in the plant [34]. This situation has been reported in studies. In one study, the effects of salicylic acid applied at different concentrations on plant growth in *Capsicum annuum* L. under in vitro conditions were investigated. 0.1 mM, 0.5 mM, and 1 mM SA concentrations were added to MS culture medium. The study concluded that low SA concentrations (0.1–0.5 mM) supported root growth, while high SA concentrations (1 mM) inhibited root growth. These findings are consistent with the results obtained in our study [66].

### Shoot length (cm)

Shoot length results are shown in Fig. 2. The NaHCO_3_ x SNP/SA interaction was found to be statistically significant (*p* ≤ 0.05). In the study, at a NaHCO_3_ concentration of 0 mM, the longest shoots were observed in the 100 µM SNP (4.25 cm) application, and the shortest shoots were observed in the 150 µM SA (1.49 cm) application. At a NaHCO_3_ concentration of 20 mM, the longest shoots were observed in the 20 mM NaHCO_3_ + 50 µM SA (2.36 cm) application, and the shortest shoots were observed in the 20 mM NaHCO_3_ + 150 µM SA (0.98 cm) application. At a concentration of 40 mM NaHCO_3_, the longest shoots were observed in the 40 mM NaHCO_3_ + 100 µM SNP (1.45 cm) application, while the shortest shoots were observed in the 40 mM NaHCO_3_ + 150 µM SA (0.77 cm) application. In our study, it was determined that a significant decrease in shoot length occurred with increasing NaHCO_3_ concentrations. It is reported that plant growth is negatively affected under alkaline stress conditions due to restrictions in nutrient uptake [67]. Alkaline conditions contain high concentrations of carbonate and bicarbonate. Furthermore, the combination of high Na^+^ content, high pH, ​​and osmotic stress disrupts cellular ion balance, causing disruptions in plant growth and development processes [68]. The 100 µM SNP application resulted in the longest shoot development under 0 mM NaHCO_3_ conditions. Low NO concentrations in plants can rapidly eliminate lipid peroxyl radicals. It can modify reactive oxygen species and their components. Thus, damage caused by reactive oxygen species is prevented, and the expression of antioxidant genes is affected [69, 70]. SNP, a nitric oxide donor, promotes cell division and elongation by replicating the effects of certain cytokinins. Although the effects vary depending on the species and the applied concentration, it supports plant growth and shoot development [25, 71, 72]. In the study, the application of 100 µM SNP increased shoot length by mitigating the negative effects of oxidative stress under high alkaline stress conditions. The findings show that SNP can support plant growth by reducing the effects of oxidative stress under certain conditions; however, it cannot completely eliminate the suppressive effect of high concentrations of alkaline stress on growth. In applications at a concentration of 20 mM NaHCO_3_, 50 µM SA showed a shoot length-enhancing effect, while 150 µM SA application at all NaHCO_3_ concentrations significantly suppressed shoot growth. SA is known as a phytohormone that regulates plant growth, development, and the plant’s response to stress factors [73]. Studies have shown that SNP and SA applied at low concentrations promote plant growth, while high concentrations lead to phytotoxic effects [74, 75]. Literature findings regarding SNP and SA applications under in vitro conditions support the results obtained from this study. The improvement of shoot length by low concentrations of SNP in Mexican lime under drought stress in in vitro conditions is consistent with the results obtained from our study [44]. Similarly, in a study investigating the effects of 50, 100, and 200 µM SA applied under in vitro conditions on shoot growth of *Stevia rebaudiana*, it was reported that the application of 50 µM SA resulted in the longest shoots, while the application of 200 µM SA reduced shoot length [76].

### Number of leaves (per plantlet)

Number of leaves results are shown in Fig. 2. The NaHCO_3_ x SNP/SA interaction was found to be statistically significant (*p* ≤ 0.05). At a NaHCO_3_ concentration of 0 mM, the highest number of leaves was observed in the 100 µM SA application (19.94 per plantlet), and the lowest number of leaves was observed in the 150 µM SNP application (13.97 per plantlet). At a NaHCO_3_ concentration of 20 mM, the highest number of leaves was observed in the 20 mM NaHCO_3_ + 50 µM SA application (20.63 per plantlet), and the lowest number of leaves was observed in the 20 mM NaHCO_3_ + 150 µM SNP application (5.33 per plantlet). At a concentration of 40 mM NaHCO_3_, the highest number of leaves was observed in the 40 mM NaHCO_3_ application (19.94 per plantlet), while the lowest number of leaves was observed in the 40 mM NaHCO_3_ + 150 µM SNP application (7.78 per plantlet). In the study, the highest number of leaves occurred in the control group at 40 mM NaHCO_3_. This can be explained by the ability of the Garnem rootstock to be grown in infertile soils and its tolerance to iron chlorosis caused by high pH [3, 77]. While low concentrations of SNP and SA applications mitigated the effects of oxidative stress, high concentrations of SNP and SA reduced the number of leaves. The highest number of leaves was obtained with SA application at 20 mM NaHCO_3_, indicating that low concentrations of SA application can increase abiotic stress tolerance. Salicylic acid is reported to contribute to the regulation of physiological processes in plants, such as improving water retention capacity, increasing the activation of antioxidant defense systems, and strengthening cell wall structure. These physiological regulations help prevent damage under stress conditions, maintain metabolic balance, and promote plant growth and development [78, 79]. Studies have reported that SA application increases number of leaves. In a study examining the effects of 50, 100, and 200 µM SA application on the number of leaves *Stevia rebaudiana* under in vitro conditions, it was reported that the low concentration of 50 µM SA resulted in the highest number of leaves, while the high concentration of 200 µM SA significantly reduced the number of leaves. These findings are consistent with the results obtained in our study [76]. It has been reported that SA applications under drought stress in vitro did not affect the number of leaves in control and mildly stressed plants of *Impatiens walleriana* L., but that 0, 1, 2 and 3 mM SA applications under high drought stress positively affected the average number of leaves, thus reducing the negative impact of drought [80].

### Number of root (per plantlet)

**Fig. 3:**
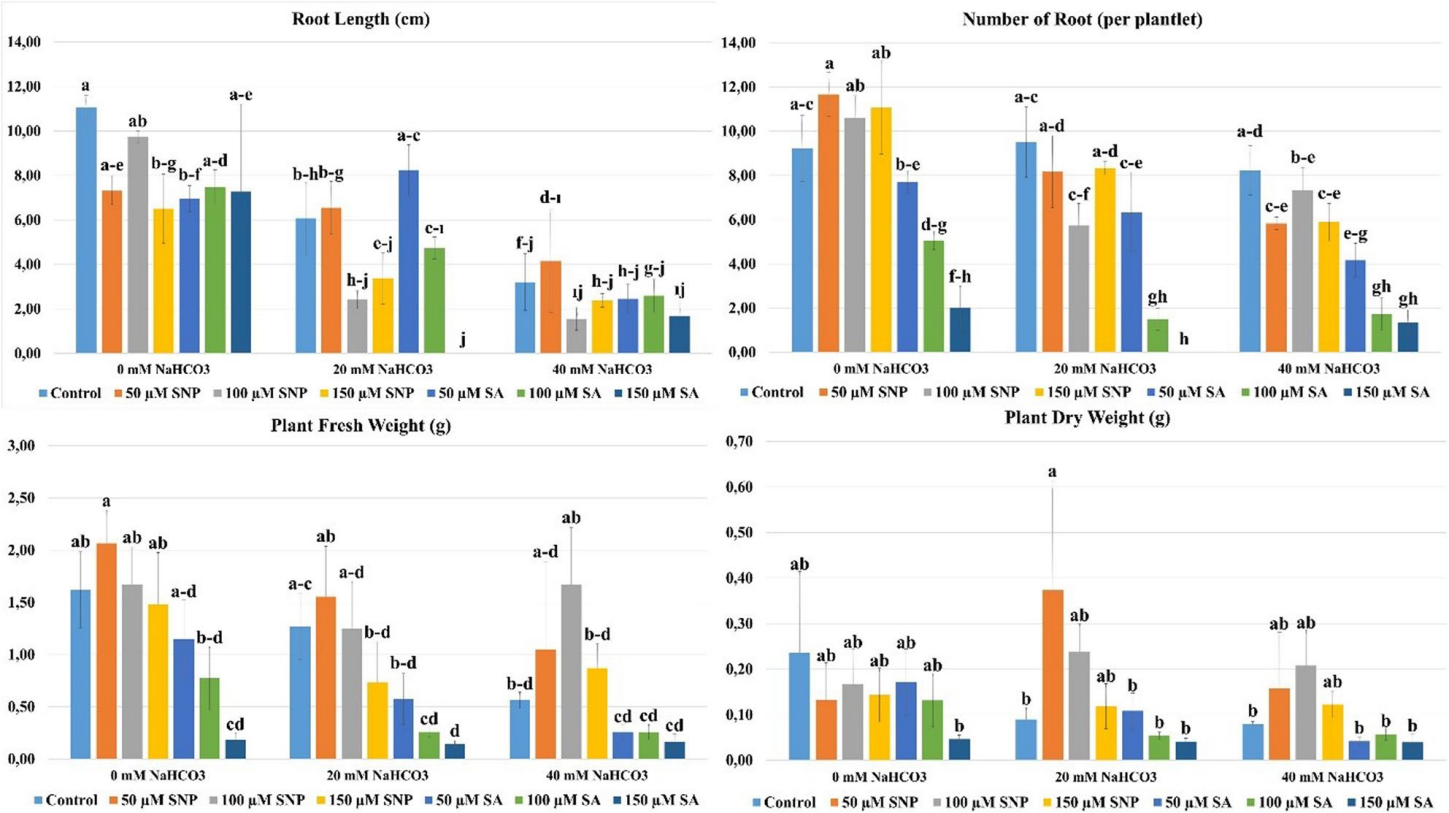
Effects of SNP and SA applications on number of root, root length, plant fresh weight and plant dry weight parameters under NaHCO_3_-induced alkaline stress conditions in vitro (p≤0.05). *The letter groupings used in the statistical analysis indicate differences among the NaHCO_3_ × SNP/SA interactions. According to Tukey’s multiple comparison test, mean values denoted by different letters differ significantly at the 5% significance level

Number of root results are shown in Fig. 3. The NaHCO_3_ x SNP/SA interaction was found to be statistically significant (*p* ≤ 0.05). At a NaHCO_3_ concentration of 0 mM, the highest number of root was observed with the 50 µM SNP (11.67 per plantlet) application, and the lowest number of root was observed with the 150 µM SA (2.00 per plantlet) application. At a NaHCO_3_ concentration of 20 mM, the highest number of root was observed with the 20 mM NaHCO_3_ (9.50 per plantlet) application, and the lowest number of root was observed with the 20 mM NaHCO_3_ + 150 µM SA (0.00 per plantlet) application. At a 40 mM NaHCO_3_ concentration, the highest number of roots occurred with the 40 mM NaHCO_3_ application (8.22 per plantlet), while the lowest number of roots occurred with the 40 mM NaHCO_3_ + 150 µM SA application (1.33 per plantlet). Among all NaHCO_3_ concentrations, the highest number of roots occurred with the 50 µM SNP concentration applied at a 0 mM NaHCO_3_ concentration. This indicates that NO can interact with auxins and cytokinins to direct differentiation and redifferentiation processes in plant cells and correlate cell division rates with these processes. In this context, it was determined that only SNP application best promoted root formation at the shoot base, providing a gradual increase in the number of adventitious and lateral roots and significantly increasing the rooting rate [25, 81, 82]. There are studies that have shown that SNP applications promote root formation under in vitro conditions. In a study conducted within this scope, it was reported that the application of 50 µM SNPs had a positive effect on rooting in three different cherry rootstocks under in vitro conditions, and that the rootstocks responded differently to SNP concentrations due to their different genotypes [83]. Similarly, in a study examining the effects of 100, 200, and 300 µM SNP applications on the vegetative development of *Aronia melanocarpa* [Michx.] Elliot under in vitro conditions, it was reported that low concentrations of SNP application increased the number of roots, while high concentrations led to a significant decrease in the number of roots [7]. In the study, it was determined that high concentrations of SA (150 µM) negatively affected the number of roots at three different NaHCO_3_ concentrations. This situation is associated with the high SA concentration causing oxidative stress and consequently increasing the production of reactive oxygen species [84, 85]. Increased oxidative stress is thought to suppress root development by reducing cell division. In this context, Sajid and Aftab [60] reported in a study conducted on three different potato varieties under salt stress in vitro that moderate SA increased the number of roots, while high SA concentrations did not show a beneficial effect; these findings support the results of our study.

### Root length (cm)

Root length results are shown in Fig. 3. The NaHCO_3_ x SNP/SA interaction was found to be statistically significant (*p* ≤ 0.05). At a concentration of 0 mM NaHCO_3_, the longest roots were found in the control treatment (11.06 cm), and the shortest roots were found in the 150 µM SNP treatment (6.51 cm). At a concentration of 20 mM NaHCO_3_, the longest roots were found in the 20 mM NaHCO_3_ + 50 µM SA treatment (8.24 cm), and the shortest roots were found in the 20 mM NaHCO_3_ + 150 µM SA treatment (0.00 cm). At a NaHCO_3_ concentration of 40 mM, the longest roots were observed in the 40 mM NaHCO_3_ + 50 µM SNP (4.16 cm) application, while the shortest roots were observed in the 40 mM NaHCO_3_ + 100 µM SNP (1.55 cm) application. Under alkaline stress conditions, root lengths decreased as NaHCO_3_ concentration increased, demonstrating a negative relationship between alkaline stress and root growth. While SNP and SA applied at low concentrations (50 µM) mitigated the negative effects of alkaline stress on root length, it was determined that high concentrations (150 µM) of SA reduced root length. It has been reported that auxins and nitric oxide (NO) synergize by promoting the formation and elongation of lateral, adventitious, and hairy roots [25, 86]. In a study based on this information, it was reported that root lengths increased in *Hyssopus officinalis* plants up to 15 µM SNP application, while decreases occurred with 20 µM SNP application [87]. The application of SA at low concentrations showed a beneficial effect under alkaline stress conditions, increasing root length. In one study, the effects of different SA (0, 0.5 and 1 mM) concentrations on two Hibiscus species (*Hibiscus moscheutos* cv. ‘Luna Red’ and *Hibiscus acetosella*) under salinity stress in vitro were investigated. The findings that root length was significantly reduced in both species under 175 and 200 mM NaCl applications, while low concentration (0.5 mM) SA application mitigated the negative effects of salinity stress and increased root length, are consistent with the results obtained in our study [88].

### Plant fresh weight and dry weight (g)

**Fig. 4:**
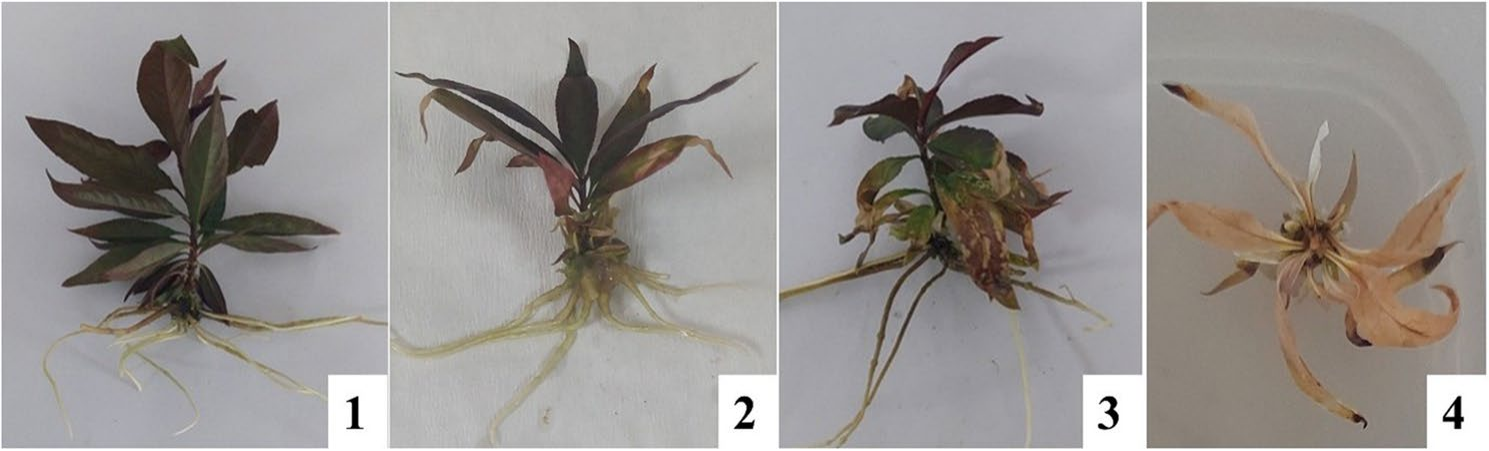
Injury index scale based on the severity of damage induced by NaHCO_3_-related alkaline stress in plants. 1: No injury, 2: Necrosis at the leaf tips and edges, 3: Necrosis throughout the leaf, 4: Dead

Plant fresh weight and dry weight results are shown in Fig. 3. The NaHCO_3_ x SNP/SA interaction was found to be statistically significant (*p* ≤ 0.05). At a 0 mM NaHCO_3_ concentration, the highest plant fresh weight was observed with 50 µM SNP (2.07 g), and the lowest plant fresh weight was observed with 150 µM SA (0.19 g). At a 0 mM NaHCO_3_ concentration, the highest plant dry weight was observed with the control (0.24 g), and the lowest plant dry weight was observed with the 150 µM SA (0.05 g) application. At a 20 mM NaHCO_3_ concentration, the highest plant fresh weight was observed with 20 mM NaHCO_3_ + 50 µM SNP (1.55 g), and the lowest plant fresh weight was observed with 20 mM NaHCO_3_ + 150 µM SA (0.14 g). At a NaHCO_3_ concentration of 20 mM, the highest plant dry weight was obtained with the 20 mM NaHCO_3_ + 50 µM SNP (0.09 g) application, and the lowest plant dry weight was obtained with the 20 mM NaHCO_3_ + 150 µM SA (0.04 g) application. At a NaHCO_3_ concentration of 40 mM, the highest plant fresh weight was obtained with the 40 mM NaHCO_3_ + 100 µM SNP (1.67 g) application, and the lowest plant fresh weight was obtained with the 40 mM NaHCO_3_ + 150 µM SA (0.17 g) application. At a NaHCO_3_ concentration of 40 mM, the highest plant dry weight was obtained with the 40 mM NaHCO_3_ + 100 µM SNP (0.21 g) application, and the lowest plant dry weight was obtained with the 40 mM NaHCO_3_ + 150 µM SA (0.04 g) application. Alkaline stress inhibits ion uptake and disrupts the ion balance of plant cells [89, 90]. Furthermore, the high pH that occurs under alkaline stress conditions causes the precipitation of various macro and micro elements, making them unavailable to the plant [91–93]. As a result, it is believed that the plant’s growth and biomass are negatively affected. In our study, it was determined that 50 and 100 µM SNP concentrations showed a corrective effect against alkaline stress, increasing plant fresh and dry weight. SNP, reduce the effect of abiotic stress on plant growth and development while also strengthening the plant’s responses to stress conditions [94, 95]. There are studies showing that SNP applications increase plant fresh and dry weight. In another study, the effects of SNP applications against drought stress in Mexican lime under in vitro conditions were investigated; it was determined that the lowest concentration of 25 µM SNP application had positive effects on shoot fresh and dry weights, thus mitigating the effects of drought stress. This finding is consistent with the results obtained in our study [44]. Similarly, in vitro conditions under salinity stress were applied to *Rubus idaeus* with 50 and 100 µM SNPs; it was reported that the application of low concentrations of SNPs increased shoot fresh and dry weights, mitigating the negative effects of salinity stress on biomass [96]. In our study, the lowest plant fresh and dry weights occurred with the application of 150 µM SA. Studies have shown that high concentrations of SA application cause cell death, negatively affecting plant development [97]. Similarly, in our study, the lowest plant fresh and dry weights were observed at high SA concentrations, which is thought to be due to the toxic effects of high-concentration SA application (Fig. 4).

### Injury index

**Fig. 5:**
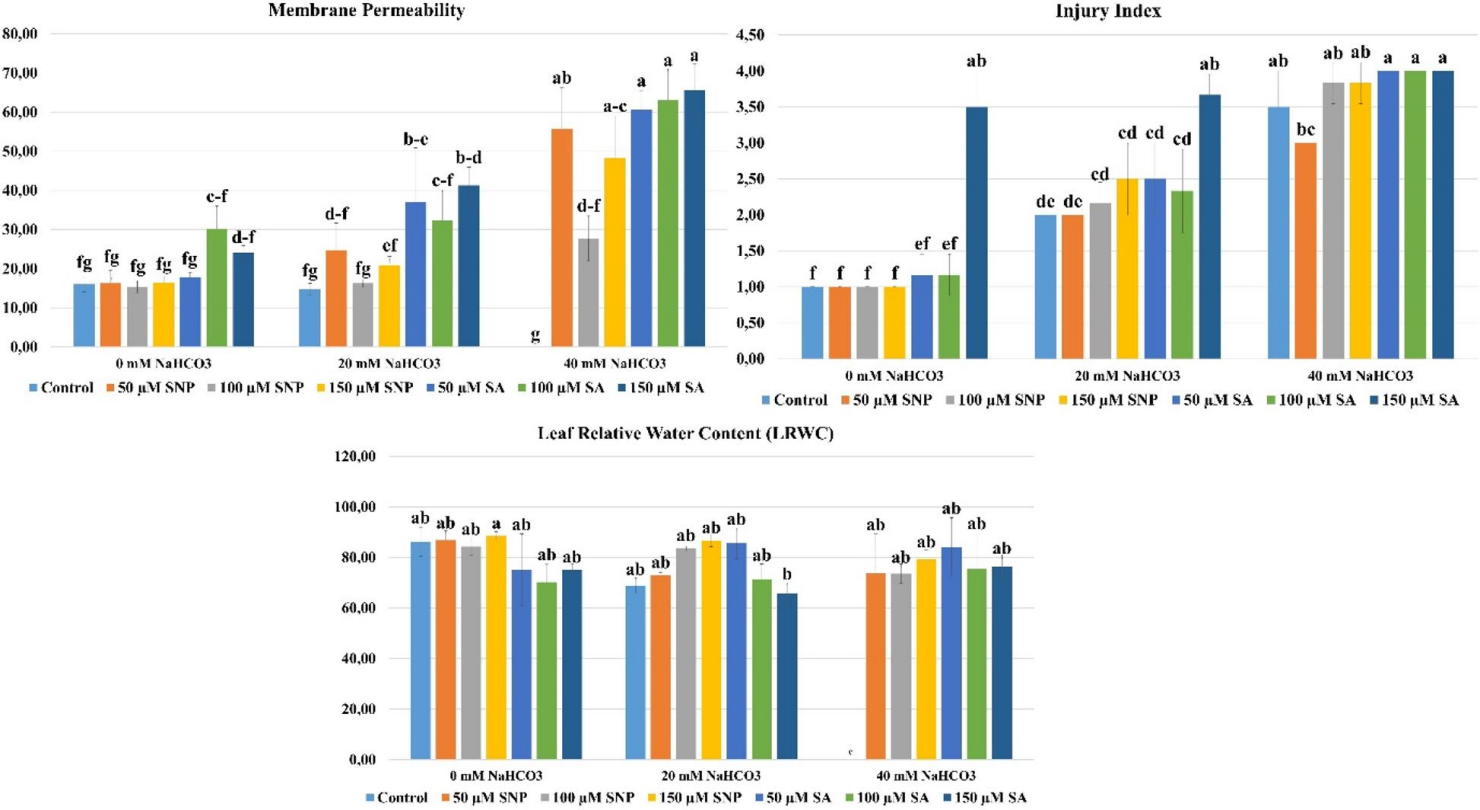
Hierarchical clustering analysis (HCA) for morphological, physiological and biochemical parameters of SNP and SA treatments applied against alkaline stress in 'Garnem' rootstock under in vitro conditions

Injury index results are shown in Fig. 4-5. The NaHCO_3_ x SNP/SA interaction was found to be statistically significant (*p* ≤ 0.05). At a concentration of 0 mM NaHCO_3_, the highest injury occurred with 150 µM SA (3.50), and the lowest injury occurred with the control, 50 µM SNP, 100 µM SNP, and 150 µM SNP (1.00) applications. In the study, at a concentration of 20 mM NaHCO_3_, the highest injury was determined with 20 mM NaHCO_3_ + 150 µM SA (3.67), and the lowest injury was determined with 20 mM NaHCO_3_ + 50 µM SNP (2.00) application. At a concentration of 40 mM NaHCO_3_, the highest injury occurred with 40 mM NaHCO3 + 50 µM SA, 40 mM NaHCO_3_ + 100 µM SA, and 40 mM NaHCO_3_ + 150 µM SA (4.00), while the lowest injury occurred with 40 mM NaHCO_3_ + 50 µM SNP (3.00). The severity of the injury increased with increasing NaHCO_3_ concentration. Injury was lowest at 0 mM NaHCO_3_ in the control and SNP applications, while the highest injury occurred with 150 µM SA application. High SA concentration caused necrosis in all parts of the plantlets’ leaves. Necrosis occurred at the leaf tips and edges at 20 mM NaHCO_3_. In the applications performed, the highest injury reached 4.00 at a concentration of 40 mM NaHCO_3_. The study determined that the application of 50 µM SNPs reduced the injury index at all three NaHCO_3_ concentrations, while high concentrations of SA increased the injury index under alkaline stress conditions. This indicates that SNPs can have a corrective effect to a certain extent under high stress conditions. NO is reported to play an important role in tolerance to stress conditions caused by oxidative damage due to its capacity to scavenge reactive intermediates and stop chain reactions resulting from its free radical properties [98].

### Membrane permeability (%)

Membrane permeability results are shown in Fig. 5. The NaHCO_3_ x SNP/SA interaction was found to be statistically significant (*p* ≤ 0.05). At a NaHCO_3_ concentration of 0 mM, the highest membrane permeability was observed with 100 µM SA (30.13%), and the lowest membrane permeability was observed with 100 µM SNP (15.43%). At a NaHCO_3_ concentration of 20 mM, the highest membrane permeability was observed with 20 mM NaHCO_3_ + 150 µM SA (41.29%), and the lowest membrane permeability was observed with 20 mM NaHCO_3_ (14.72%). At a NaHCO_3_ concentration of 40 mM, the highest membrane permeability was observed with 40 mM NaHCO_3_ + 150 µM SA (65.61%), while the lowest membrane permeability was observed with 40 mM NaHCO_3_ (0.00%). Membrane permeability refers to an ion imbalance that develops due to intracellular and extracellular osmotic imbalance [99]. Under alkaline stress conditions, the accumulation of excessively reactive oxygen species causes lipid peroxidation in the cell membrane, increasing membrane permeability [100, 101]. It was determined that increasing NaHCO_3_ concentrations increased membrane permeability. While 100 µM SNP application minimized membrane damage at all NaHCO_3_ concentrations, 150 µM SA applications caused the most membrane damage. This finding demonstrates that SNP application has a protective effect on the cell membrane at low concentrations. Nitric oxide is an important signaling molecule in plant defense systems. It increases the activity of antioxidant enzymes, thiols, and compatible osmolytes. Thus, it protects plants against stress conditions caused by salinity, drought, and heavy metals, while preventing structural damage to the cell membrane, ion and metal-induced toxicity, osmotic stress, lipid peroxidation, and excessive accumulation of reactive oxygen species [102]. In this context, a study showing that SNP applied to Mexican lime under in vitro conditions to improve the harmful effects of drought stress reduced membrane permeability supports our study [44]. In our study, it was determined that all SA applications caused an increase in membrane permeability. It is stated that the positive effects of salicylic acid on plants against abiotic and biotic stress conditions generally occur when applied at low doses. Conversely, it has been reported that SA applied in high concentrations can cause oxidative damage and negatively affect stress tolerance [27, 103].

### Leaf relative water content (LRWC)

The results regarding leaf relative water content are shown in Fig. 5. The NaHCO_3_ x SNP/SA interaction was found to be statistically significant (*p* ≤ 0.05). At a 0 mM NaHCO_3_ concentration, the highest leaf relative water content was found in the 150 µM SNP (88.54%) application, and the lowest leaf relative water content was found in the 100 µM SA (70.22%) application. At a 20 mM NaHCO_3_ concentration, the highest leaf relative water content was found in the 20 mM NaHCO_3_ + 150 µM SNP (86.72%) application, and the lowest leaf relative water content was found in the 20 mM NaHCO_3_ + 150 µM SA (65.71%) application. At a 40 mM NaHCO_3_ concentration, the highest leaf relative water content was observed with 40 mM NaHCO_3_ + 50 µM SA (84.15%), while the lowest leaf relative water content was observed with 40 mM NaHCO_3_ (0.00%). No fresh leaves were found for LRWC determination due to plantlet damage at the 40 mM NaHCO_3_ concentration without SNP and SA application. Therefore, LRWC values ​​were recorded as 0.00% to indicate complete tissue desiccation instead of a measurable water status. Leaf relative water content decreased with increasing NaHCO_3_ concentration. Plant water content is important for maintaining turgor and for the efficient uptake of mineral nutrients by the roots. Reductions in plant water content cause cells to shrink due to loss of turgor. In this case, the plant tries to balance cell volume by activating physiological defense mechanisms to maintain osmotic balance [4, 104]. Based on the data obtained from our study, it was determined that SNP application increased the relative water content of the leaves of plantlets under alkaline stress conditions. SNP is an important signaling molecule that mediates the regulation of physiological processes in plants. It prevents plant water loss by regulating stomatal movements under abiotic stress conditions [105–107]. This situation is confirmed by the result that SNP applications applied against alkaline stress increased the relative water content of the leaves in our study and is supported by other studies [96]. In our study, SA applications in the unstressed (0 mM NaHCO_3_) group decreased the relative water content of the leaves compared to the control, while SA applications under alkaline stress conditions yielded positive results and increased the relative water content of the leaves. SA is a naturally occurring phenolic compound. In addition to regulating plant physiological processes, it also plays an important role in stomatal movements and plant respiration [30, 108, 109]. In our study, it is thought that the application of 50 µM SA at a high concentration (40 mM NaHCO_3_) under alkaline stress conditions increased the relative water content of the leaf and prevented water loss by causing the plant stomata to close under stress conditions.

### Chlorophyll a, b and total concentrations

Results related to chlorophyll a, b, and total chlorophyll content are presented in Table 2. The NaHCO_3_ x SNP/SA interaction was found to be statistically significant (*p* ≤ 0.05). At a 0 mM NaHCO_3_ concentration, the highest chlorophyll a (4.87), chlorophyll b (6.06), and total chlorophyll content (10.92) were observed in the control group, while the lowest chlorophyll a (2.91), chlorophyll b (3.66), and total chlorophyll content (6.56) occurred in the 0 mM NaHCO_3_ + 150 µM SA application. At a concentration of 20 mM NaHCO_3_, the highest chlorophyll a (3.77), chlorophyll b (4.57), and total chlorophyll content (8.34) were observed in the 20 mM NaHCO_3_ + 50 µM SNP application, while the lowest chlorophyll a (0.00), chlorophyll b (0.00), and total chlorophyll content (0.00) were observed in the 20 mM NaHCO_3_ + 150 µM SA application. At a concentration of 40 mM NaHCO_3_, the highest chlorophyll a (3.94), chlorophyll b (4.85), and total chlorophyll content (8.79) were determined in the 40 mM NaHCO_3_ + 50 µM SNP application, while the lowest chlorophyll a, b, and total chlorophyll content occurred in the 40 mM NaHCO_3_, 40 mM NaHCO_3_ + 100 µM SNP, 40 mM NaHCO_3_ + 150 µM SNP, 40 mM NaHCO_3_ + 50 µM SA, 40 mM NaHCO_3_ + 100 µM SA, and 40 mM NaHCO_3_ + 150 µM SA (0.00) applications. In these applications, the plantlets were damaged due to alkaline stress, and chlorophyll a, b, and total chlorophyll content could not be measured. Compared to the non-alkaline stress (0 mM NaHCO_3_) application, increasing the NaHCO_3_ concentration resulted in a significant decrease in chlorophyll a, b, and total chlorophyll content. Photosynthesis is the main source of carbon and energy necessary for plant growth and development [110, 111]. Studies have shown that salinity and alkaline stress can lead to a decrease in photosynthesis, which in turn can cause reductions in photosynthetic rate, stomatal conductivity, transpiration rate, and photosynthetic pigment levels. Alkaline stress is more damaging to plant photosynthesis than salinity stress [112–115]. High concentrations of NaHCO_3_ in the soil increase soil pH. This leads to high sodium levels, low water infiltration capacity, and high bicarbonate levels in the soil. High bicarbonate ion concentrations in the plant root zone inhibit the uptake of many micronutrients, especially iron (Fe). This situation is considered the primary factor in the development of chlorosis in plants under alkaline conditions [116, 117]. In our study, the pigment loss observed at 40 mM NaHCO_3_ is due to ion toxicity. In our study, it was determined that SNP applications increased chlorophyll content, while SA applications decreased chlorophyll content with increasing concentration. Low concentration (50 µM SNP) application increased chlorophyll content against alkaline stress. NO prevents the deterioration of the chloroplast membrane structure under stress conditions, thus preserving the content of photosynthetic pigments [118, 119]. Salicylic acid can cause stress for plants in some cases and can negatively affect photosynthesis processes, especially at concentrations above a certain threshold. Furthermore, the concentration range in which this compound is effective can vary significantly depending on the plant species, application method, duration, and environmental factors [120]. A study investigated the effects of SA application (1 mM, 0.5 mM, and 0.1 mM) on the plant growth of *Capsicum annuum* L. under in vitro conditions. The study reported that 0.1 mM SA application increased total chlorophyll content, while higher SA concentrations resulted in chlorophyll a content similar to or lower than the control group. These findings are consistent with our study’s finding that increasing SA concentrations decreased chlorophyll content [66].

**Table 2:** Effect of SNP and SA applications on chlorophyll content under NaHCO_3_ stress

Treatments	Chlorophyll a	Chlorophyll b	Total chlorophyll
0 mM NaHCO_3_	4.87 ± 0.26^a^	6.06 ± 0.37^a^	10.92 ± 0.61^a^
20 mM NaHCO_3_	3.39 ± 0.58^b^	4.12 ± 0.75^bc^	7.51 ± 1.33^bc^
40 mM NaHCO_3_	0.00 ± 0.00^d^	0.00 ± 0.00^e^	0.00 ± 0.00^e^
0 mM NaHCO_3_ + 50 µM SNP	4.29 ± 0.73^ab^	5.10 ± 0.87^abc^	9.39 ± 1.60^abc^
0 mM NaHCO_3_ + 100 µM SNP	4.85 ± 0.55^a^	5.75 ± 0.65^ab^	10.60 ± 1.21^ab^
0 mM NaHCO_3_ + 150 µM SNP	3.90 ± 0.55^ab^	4.70 ± 0.69^abc^	8.60 ± 1.24^abc^
20 mM NaHCO_3_ + 50 µM SNP	3.78 ± 0.53^ab^	4.57 ± 0.65^abc^	8.34 ± 1.18^abc^
20 mM NaHCO_3_ + 100 µM SNP	3.23 ± 0.40^bc^	4.11 ± 0.41^bc^	7.34 ± 0.80^c^
20 mM NaHCO_3_ + 150 µM SNP	3.50 ± 0.39^ab^	4.52 ± 0.44^abc^	8.02 ± 0.82^abc^
40 mM NaHCO_3_ + 50 µM SNP	3.94 ± 0.17^ab^	4.85 ± 0.14^abc^	8.79 ± 0.28^abc^
40 mM NaHCO_3_ + 100 µM SNP	0.00 ± 0.00^d^	0.00 ± 0.00^e^	0.00 ± 0.00^e^
40 mM NaHCO_3_ + 150 µM SNP	0.00 ± 0.00^d^	0.00 ± 0.00^e^	0.00 ± 0.00^e^
0 mM NaHCO_3_ + 50 µM SA	3.80 ± 0.59^ab^	4.61 ± 0.72^abc^	8.41 ± 1.32^abc^
0 mM NaHCO_3_ + 100 µM SA	3.63 ± 0.19^ab^	4.39 ± 0.23^abc^	8.01 ± 0.43^abc^
0 mM NaHCO_3_ + 150 µM SA	2.91 ± 1.26^bc^	3.66 ± 1.63^cd^	6.56 ± 2.88^cd^
20 mM NaHCO_3_ + 50 µM SA	3.56 ± 0.18^ab^	4.38 ± 0.22^abc^	7.95 ± 0.40^abc^
20 mM NaHCO_3_ + 100 µM SA	1.84 ± 0.37^c^	2.28 ± 0.41^d^	4.12 ± 0.78^d^
20 mM NaHCO_3_ + 150 µM SA	0.00 ± 0.00^d^	0.00 ± 0.00^e^	0.00 ± 0.00^e^
40 mM NaHCO_3_ + 50 µM SA	0.00 ± 0.00^d^	0.00 ± 0.00^e^	0.00 ± 0.00^e^
40 mM NaHCO_3_ + 100 µM SA	0.00 ± 0.00^d^	0.00 ± 0.00^e^	0.00 ± 0.00^e^
40 mM NaHCO_3_ + 150 µM SA	0.00 ± 0.00^d^	0.00 ± 0.00^e^	0.00 ± 0.00^e^

*The letter groupings used in the statistical analysis indicate differences among the NaHCO_3_× SNP/SA interactions. According to Tukey’s multiple comparison test, mean values denoted by different letters differ significantly at the 5% significance level

#### Hydrogen peroxide (H_2_O_2_)

The results related to hydrogen peroxide levels are presented in Table 3. The NaHCO_3_ x SNP/SA interaction was found to be statistically significant (*p* ≤ 0.05). Hydrogen peroxide levels are quite low at 0 mM NaHCO_3_ concentration. At this concentration, the highest hydrogen peroxide level occurred in the 150 µM SA (4.37) application, and the lowest hydrogen peroxide level occurred in the control and 150 µM SNP (2.84) applications. At a 20 mM NaHCO_3_ concentration, the highest hydrogen peroxide level occurred in the 20 mM NaHCO_3_ (94.07) application, and the lowest hydrogen peroxide level occurred in the 20 NaHCO_3_ + 150 µM SA (0.00) application. At this concentration (20 mM NaHCO_3_ + 150 µM SA), the lowest hydrogen peroxide level resulted from complete damage to the plantlets. The effect of alkaline stress at a concentration of 20 mM NaHCO_3_ was reduced by the application of 150 µM SNP. At high alkaline stress concentrations, the plantlets were damaged due to damage caused by alkaline stress, and measurements could not be taken to determine the amount of hydrogen peroxide. At this concentration, the amount of hydrogen peroxide was only measured in the application of 40 mM NaHCO_3_ + 50 µM SNP (78.31). H_2_O_2_ levels increased under alkaline stress conditions. H_2_O_2_ is naturally present in plants under normal conditions and produces reactive oxygen species such as superoxide anions (O_2_^−^) and hydrogen peroxide (H_2_O_2_) in response to environmental stressors. These reactive oxygen species act as important cellular signaling molecules in the regulation of stress tolerance at low concentrations. However, at high concentrations, they cause significant oxidative damage by damaging cell membranes, proteins, RNA, and DNA. It can also result in very advanced cell deaths [121–127]. In our study, it was determined that SNP applications reduced the amount of H_2_O_2_ caused by alkaline stress. This result shows that SNPs increase the plant’s tolerance to abiotic stress conditions even at low concentrations. Furthermore, SNPs, which are nitric oxide donors, reduce active oxygen accumulation by increasing the activity of ROS scavenging enzymes [21, 128]. Studies have shown that SNP applications reduce the amount of H_2_O_2_. In a study conducted under in vitro conditions, it was found that PEG-induced drought stress increased H_2_O_2_ accumulation in the leaves of *Allium hirtifolium* plantlets, causing oxidative stress; among the applied SNP concentrations, it was reported that a concentration of 40 µM significantly reduced H_2_O_2_ accumulation, thereby mitigating the negative effects of stress [129].

**Table 3:** Effect of SNP and SA applications on MDA and H_2_O_2_ parameters under NaHCO_3_ stress

Treatments	H_2_O_2_ (µmol g^− 1^ FW)	MDA (µmol g^− 1^ FW)
0 mM NaHCO_3_	2.84 ± 1.00^h^	6.32 ± 1.00^d^
20 mM NaHCO_3_	94.07 ± 1.00^a^	16.71 ± 1.00^a^
40 mM NaHCO_3_	0.00 ± 0.00^ı^	0.00 ± 0.00^e^
0 mM NaHCO_3_ + 50 µM SNP	3.61 ± 1.00^h^	6.52 ± 1.00^d^
0 mM NaHCO_3_ + 100 µM SNP	3.35 ± 1.00^h^	6.39 ± 1.00^d^
0 mM NaHCO_3_ + 150 µM SNP	2.84 ± 1.00^h^	6.26 ± 1.00^d^
20 mM NaHCO_3_ + 50 µM SNP	69.93 ± 1.00^e^	15.48 ± 1.00^ab^
20 mM NaHCO_3_ + 100 µM SNP	62.81 ± 1.00^f^	13.23 ± 1.00^b^
20 mM NaHCO_3_ + 150 µM SNP	54.43 ± 1.00^g^	10.65 ± 1.00^c^
40 mM NaHCO_3_ + 50 µM SNP	78.31 ± 1.00^c^	15.45 ± 1.00^ab^
40 mM NaHCO_3_ + 100 µM SNP	0.00 ± 0.00 ^ı^	0.00 ± 0.00^e^
40 mM NaHCO_3_ + 150 µM SNP	0.00 ± 0.00^ı^	0.00 ± 0.00^e^
0 mM NaHCO_3_ + 50 µM SA	4.37 ± 1.00^h^	6.13 ± 1.00^d^
0 mM NaHCO_3_ + 100 µM SA	3.86 ± 1.00^h^	4.87 ± 1.00^d^
0 mM NaHCO_3_ + 150 µM SA	4.37 ± 1.00^h^	5.81 ± 1.00^d^
20 mM NaHCO_3_ + 50 µM SA	83.91 ± 1.00^b^	15.35 ± 1.00^ab^
20 mM NaHCO_3_ + 100 µM SA	73.23 ± 1.00^d^	14.65 ± 1.00^ab^
20 mM NaHCO_3_ + 150 µM SA	0.00 ± 0.00^ı^	0.00 ± 0.00^e^
40 mM NaHCO_3_ + 50 µM SA	0.00 ± 0.00^ı^	0.00 ± 0.00^e^
40 mM NaHCO_3_ + 100 µM SA	0.00 ± 0.00^ı^	0.00 ± 0.00^e^
40 mM NaHCO_3_ + 150 µM SA	0.00 ± 0.00^ı^	0.00 ± 0.00^e^

*MDA* Malondialdehyde, *H*_2_*O*_2_ Hydrogen peroxide

^*^The letter groupings used in the statistical analysis indicate differences among the NaHCO_3_ × SNP/SA interactions. According to Tukey’s multiple comparison test, mean values denoted by different letters differ significantly at the 5% significance level

### Malondialdehyde (MDA)

The results related to malondialdehyde content are presented in Table 3. The NaHCO_3_ x SNP/SA interaction was found to be statistically significant (*p* ≤ 0.05). MDA levels were quite low at 0 mM NaHCO_3_ concentration. At this concentration, the highest MDA amount was observed in the 50 µM SNP (6.52) application, and the lowest amount in the 100 µM SA (4.87) application. At 20 mM concentration, the highest MDA amount was found in the 20 mM NaHCO_3_ (16.71) application, and the lowest amount in the 20 mM NaHCO_3_ + 150 µM SA (0.00) application. The result obtained from the application where the lowest MDA amount occurred (20 mM NaHCO_3_ + 150 µM SA) is due to the complete damage of the plantlets. In the study, the effect of alkaline stress at 20 mM NaHCO_3_ was reduced by the application of 150 µM SNP. At the highest alkaline stress concentration (40 mM NaHCO_3_), the plantlets were damaged due to alkaline stress and MDA levels could not be measured. At this concentration, MDA levels were measured only in the application of 40 mM NaHCO_3_ + 50 µM SNP (15.45). MDA is one of the end products formed as a result of the peroxidation of polyunsaturated fatty acids in plant cells, and thus stands out as a reliable biochemical marker in the detection of oxidative damage at the cellular level. The more damage plants experience under biotic and abiotic stress conditions, the higher the amount of MDA [130, 131]. Under alkaline stress conditions, nutrient uptake is hampered by high pH, ​​leading to increased oxidative stress throughout plant growth. It also causes the accumulation of significant levels of reactive oxygen species, leading to oxidative damage to cellular proteins, lipids, and DNA [132]. In our study, MDA levels increased as alkaline stress concentrations increased. SNP applications at a concentration of 20 mM NaHCO_3_ yielded positive results. As the SNP concentration increased, a gradual decrease in MDA levels occurred. At a concentration of 40 mM NaHCO_3_, MDA levels could not be measured due to damage to the plantlets from alkaline stress. Measurements were only taken at this concentration with a 50 µM SNP application. These findings demonstrate that low-concentration SNP application under high alkaline stress conditions protects plantlets against damage caused by oxidative stress. This is because SNPs are signaling molecules that increase the tolerance of plants to abiotic stress conditions. SNP applications reduce active oxygen deposition in plants by increasing the activity of ROS scavenging enzymes [21, 128]. Based on this mechanism, the fact that SNPs limit oxidative damage by increasing ROS scavenging enzyme activities is supported by the reduction of MDA levels by SNP applications in our study. Studies have shown that SNPs applied under abiotic stress conditions reduce MDA levels, and these findings support our study [44, 129, 133]. A 100 µM concentration of salicylic acid applications reduced MDA levels compared to other SA concentrations. However, in our study, the negative results at high concentrations under alkaline stress conditions are thought to be due to its toxic effects. Salicylic acid, a signaling molecule, is a phenolic compound. SA enhances enzymatic antioxidant activity and reduces the production of reactive oxygen species. It plays an important role in regulating plant growth and development and in their responses to stress conditions. It also regulates important physiological processes in plants, such as the antioxidant defense system and plant-water relations under stress conditions [27, 134, 135].

### General evaluation of morphological, physiological and biochemical responses

**Fig. 6:**
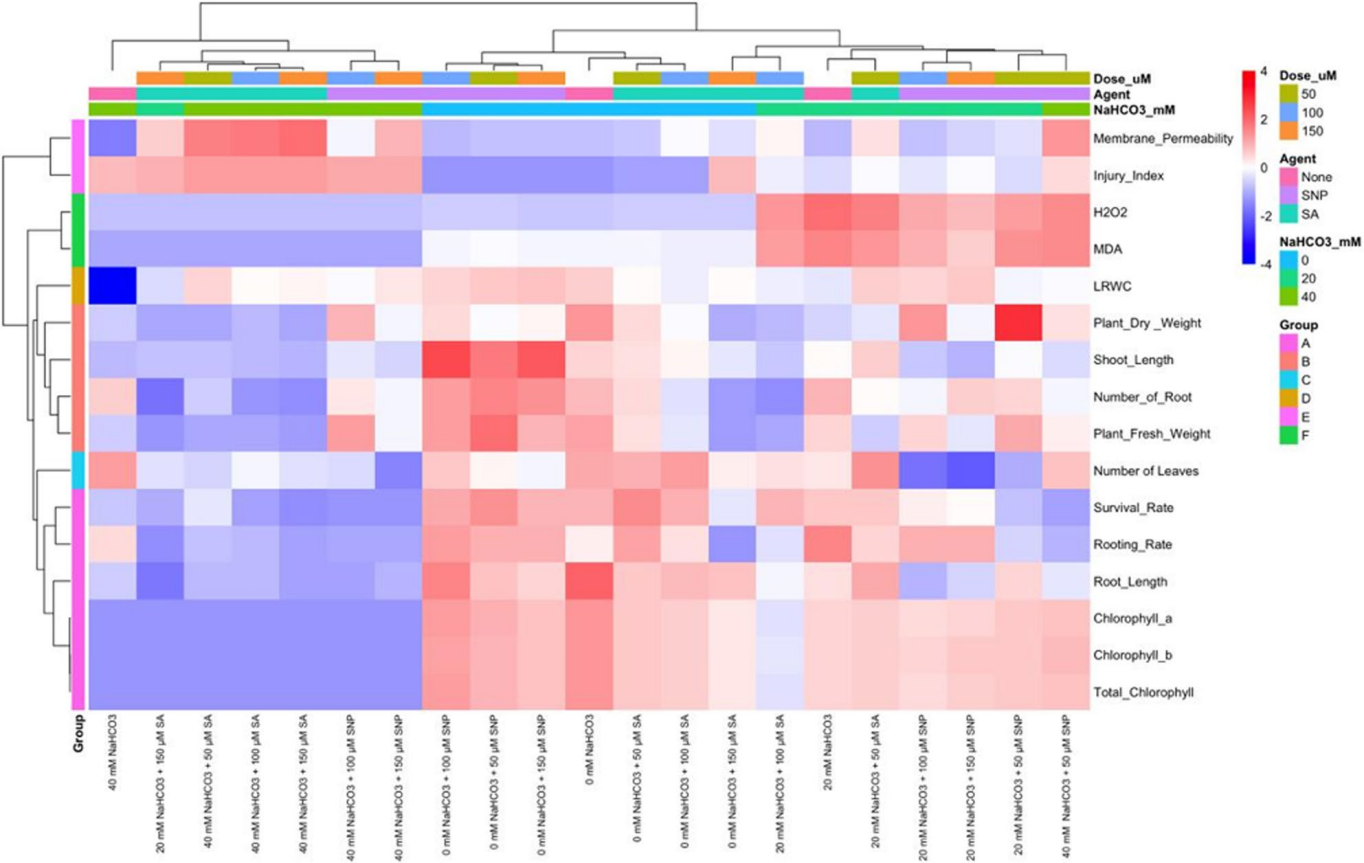
Hierarchical clustering analysis (HCA) for morphological, physiological and biochemical parameters of SNP and SA treatments applied against alkaline stress in 'Garnem' rootstock under in vitro conditions

**Fig. 7:**
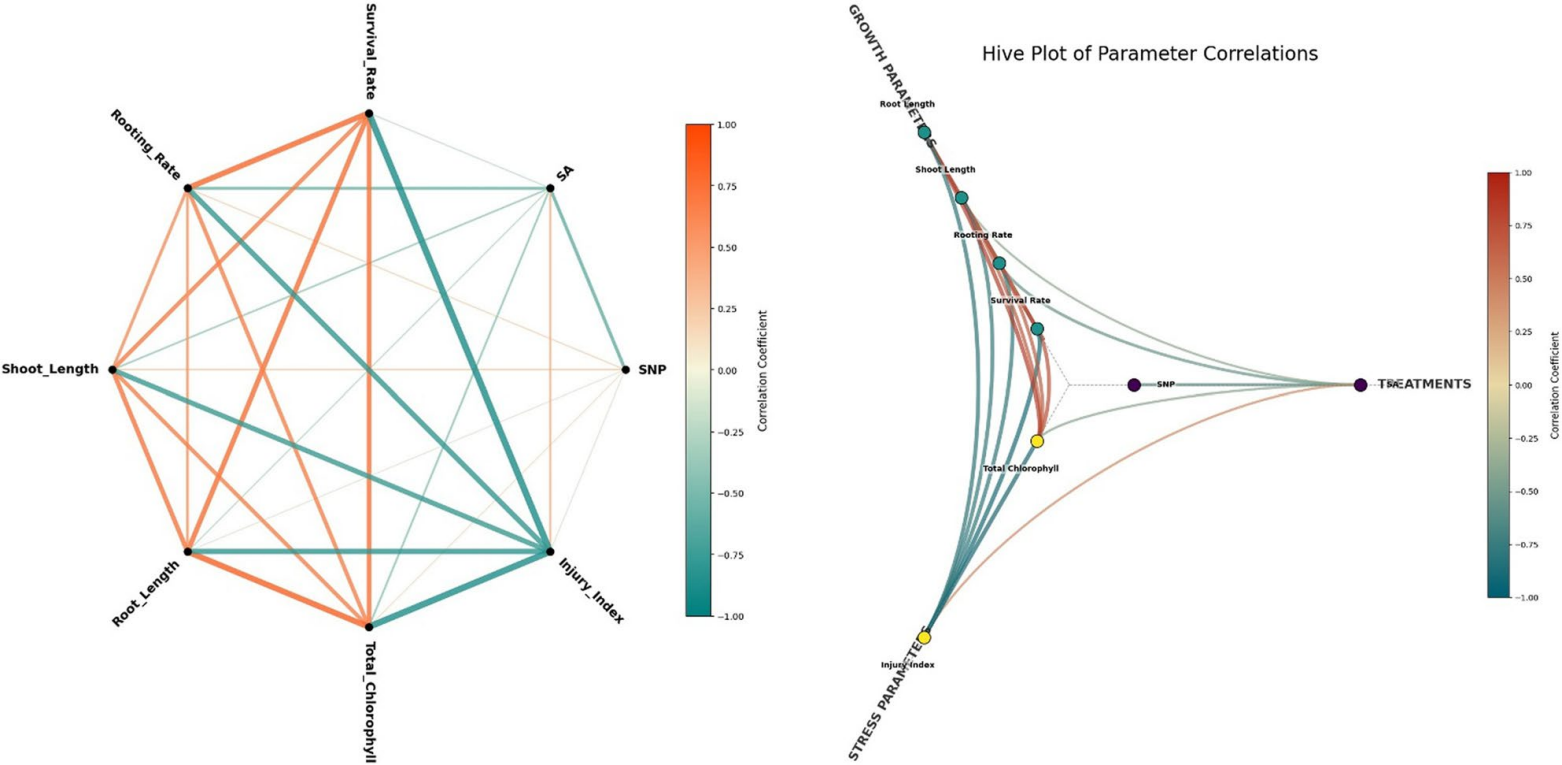
Network correlation analysis for morphological, physiological and biochemical parameters of SNP and SA treatments applied against alkaline stress in 'Garnem' rootstock under in vitro conditions

The hierarchical clustering and network correlation analyses obtained in this study reveal that the morphological and physiological responses of Garnem rootstock under alkaline stress have a complex and multidimensional structure (Fig. 6 and 7). The clustering of growth and photosynthetic capacity-related parameters (shoot and root length, survival and rooting rate, chlorophyll content) in HCA results indicates that alkaline stress has a suppressive effect on plant development, and these findings are consistent with previous studies reporting growth inhibition under salinity and alkaline stress [136]. In contrast, the concentration of stress and cellular damage indicators such as membrane permeability, injury index, H_2_O_2_, and MDA in distinct clusters supports the idea that oxidative stress is one of the key limiting factors under alkaline stress conditions [137, 138].

Network correlation analysis revealed strong positive relationships between growth and photosynthetic parameters, indicating that plant performance is shaped by the co-regulation of these characteristics (Fig. 7). In contrast, the significant negative correlations between oxidative stress markers and growth parameters suggest that under stressful conditions, plants shift resources from growth to cellular protection and stress tolerance. This is consistent with the growth–defense trade-off model [139, 140]. Similarly, the observed decreases in chlorophyll content with increasing oxidative stress are consistent with previous findings reporting that alkaline stress has disruptive effects on the photosynthetic apparatus [141, 142]. The relational patterns regarding SNP and SA applications suggest that these signaling molecules may modulate stress responses in different ways. The more stable relationship between low-dose SNP applications and growth and photosynthetic parameters is consistent with the literature reporting that nitric oxide may contribute to maintaining cellular redox balance under stress conditions [143]. In contrast, the clustering of SA applications with stress markers under high alkaline stress suggests that the effects of salicylic acid may vary depending on stress intensity and dose, and may have limiting effects on growth under certain conditions [27]. However, these multivariate analyses do not directly reveal causal mechanisms; they only identify relational patterns between parameters. Therefore, the results of the HCA and network analyses provide a supportive and integrative framework for understanding the physiological responses of the Garnem rootstock under alkaline stress.

## Conclusion

This study revealed that alkaline stress induced by NaHCO_3_, applied under in vitro conditions, caused significant negative effects on the morphological, physiological, and biochemical characteristics of Garnem (*Prunus dulcis* × *Prunus persica*) rootstock plantlets. Increasing alkaline stress concentrations led to significant reductions in plantlet survival and rooting rates, shoot and root length, number of leaves, plant fresh weight, and dry weight. Furthermore, an increase in membrane permeability, a high injury index, increased H_2_O_2_ and MDA levels, and decreases in chlorophyll a, b, and total chlorophyll content were demonstrated. In the study, SNP application effectively mitigated oxidative stress-induced damage depending on the concentration. 50 and 100 µM SNP applications improved survival and rooting rates, and increased shoot length, root length, plant fresh weight, and dry weight at 20 mM and 40 mM NaHCO_3_ concentrations. It provided protection in terms of photosynthetic pigment levels. It was shown that membrane permeability and injury index values ​​were significantly reduced, while H_2_O_2_ and MDA accumulation was significantly suppressed, improving membrane integrity and reducing oxidative damage. The protective effect of low concentration (50 µM) SNP application at 40 mM NaHCO_3_ concentration revealed that SNP has a strong potential against alkaline stress in Garnem rootstock. Low concentrations of SA (50–100 µM) mitigated the effects of alkaline stress by improving survival rate, number of leaves, leaf relative water content, and some growth parameters at 20 mM NaHCO_3_ concentration. However, the highest concentration of SA application (150 µM) consistently showed phytotoxic effects at all NaHCO_3_ concentrations, leading to decreases in growth performance, complete inhibition of rooting in some applications, and increased membrane damage and injury index. At a concentration of 40 mM NaHCO_3_, SA applications failed to provide protection and intensified alkaline stress-related damage. Based on these findings, it was determined that in vitro, SNP and SA applications under NaHCO_3_-induced alkaline stress in Garnem rootstock were effective in mitigating the negative effects of alkaline stress on different parameters.

## Electronic Supplementary Material

Below is the link to the electronic supplementary material.

Supplementary Material 1

Supplementary Material 2

Supplementary Material 3

Supplementary Material 4

Supplementary Material 5

Supplementary Material 6

## Data Availability

The data supporting the findings of this study are available from the corresponding author.

## References

[1] Gregory PJ, Atkinson CJ, Bengough AG, Else MA, Fernández-Fernández F, Harrison RJ, Schmidt S. Contributions of roots and rootstocks to sustainable, intensified crop production. J Exp Bot. 2013;64(5):1209–22. 23378378 10.1093/jxb/ers385

[2] Roberto SR, Novello V, Fazio G. New rootstocks for fruit crops: breeding programs, current use, future potential, challenges and alternative strategies. Front Plant Sci. 2022;13:878863. 35401619 10.3389/fpls.2022.878863 PMC8986155

[3] Felipe AJ. Felinem’, ‘Garnem’, and ‘Monegro’almond× peach hybrid rootstocks. HortScience. 2009;44(1):196–7.

[4] İpek M. In vitro şartlarda Garnem ve Myrobolan 29 C anaçlarının kurak stresine karşı tepkilerinin belirlenmesi. PhD thesis, Selçuk University, Institute of Science, Department of Horticulture, Konya, Türkiye. 2015. p.142.

[5] Ak BE, Ekinci H, Dikmetaş doğan B, Can ZR, Şaşkin N. Badem Yetiştiriciliğinde Kullanılan Bazı Anaçlar Ve Özellikleri (Altıncı Bölüm). Modern Badem Yetiştiriciliği (: Eds: Prof. Dr. Bekir Erol AK ve Doç. Dr. Mine PAKYÜREK), İksad Yayınevi, Ankara, 2023;141–63. (ISBN: 978-625-367-591-2), s. 10.5281/zenodo.10456013.

[6] AkBE, Yaman A. Some traits of different almond rootstocks and relationships between rootstocks and cultivars in almonds. Proceeding Book of 5th International Conference on Scientific and Academic Research (5- ICSAR 2024), December 23-24, Konya, Turkey. 2024:1077–83. (ISBN: 978-625-5954-26-8). https://www.icsarconf.com/.

[7] Ekinci H, Saskin N, Ak BE, Dogan BD. Effects of different healing agents on acclimatization success of in vitro rooted Garnem (*Prunus dulcis*× *Prunus persica*) rootstock. In Vitro Cellular & Developmental Biology-Plant. 2024;60(3):309–17.

[8] BabaoğluM, Gürel E, Özcan S. Bitki Biyoteknolojisi, Selçuk Üniversitesi Basımevi, 2002;374. https://yayinevi.selcuk.edu.tr/index.php/su/catalog/book/56.

[9] Ak BE. The Importance of in vitro micropropagation of fruit crops. In 1st International GAP agriculture & livestock congress. Proceedings Book 25-27 April, Sanliurfa/Türkiye. 2018;716–723. (ISBN 978-975-7113-65-2)

[10] Gupta N, Jain V, Joseph MR, Devi S. A review on micropropagation culture method. Asian J Pharm Res Dev. 2020;8(1):86–93.

[11] SaskinN, Ak BE, Ekinci H. In: Kirca L, Bak T, Güler E, Dogru Çokran B, Kılıç D, editors. The usage of node culture in vitro conditions. 5 ed. Denizli: International Agriculture Congress; 2022. p. 90–9. https://utak.azimder.org.tr.

[12] Wijerathna-Yapa A, Hiti-Bandaralage J. Tissue culture—a sustainable approach to explore plant stresses. Life. 2023;13(3):780. 36983935 10.3390/life13030780 PMC10057563

[13] Oster JD, Shainberg I, Abrol IP. Reclamation of salt-affected soils. Agricultural Drain. 1999;38:659–91.

[14] Zhang H, Liu XL, Zhang RX, Yuan HY, Wang MM, Yang HY, et al. Root damage under alkaline stress is associated with reactive oxygen species accumulation in rice (*Oryza sativa* L.). Front Plant Sci. 2017;8:1580. 28943882 10.3389/fpls.2017.01580 PMC5596797

[15] Kaiwen G, Zisong X, Yuze H, Qi S, Yue W, Yanhui C, et al. Effects of salt concentration, pH, and their interaction on plant growth, nutrient uptake, and photochemistry of alfalfa (*Medicago sativa*) leaves. Plant Signal Behav. 2020;15(12):1832373. 33073686 10.1080/15592324.2020.1832373 PMC7671061

[16] Fang S, Hou X, Liang X. Response mechanisms of plants under saline-alkali stress. Front Plant Sci. 2021;12:667458. 34149764 10.3389/fpls.2021.667458 PMC8213028

[17] Liu D, Ma Y, Rui M, Lv X, Chen R, Chen X, et al. Is high pH the key factor of alkali stress on plant growth and physiology? A case study with wheat (*Triticum aestivum* L.) seedlings. Agronomy. 2022;12(8):1820.

[18] Sharma M, Tisarum R, Kohli RK, Batish DR, Cha-Um S, Singh HP. Inroads into saline-alkaline stress response in plants: unravelling morphological, physiological, biochemical, and molecular mechanisms. Planta. 2024;259(6):130. 38647733 10.1007/s00425-024-04368-4

[19] Pérez-Labrada F, Espinoza-Acosta L, Bárcenas-Santana J, García-León D, E, López-Pérez C. M. Underlying mechanisms of action to improve plant growth and fruit quality in crops under alkaline stress. IntechOpen. 2024. 10.5772/intechopen.114335.

[20] Kaur G, Tak Y, Asthir B. Salicylic acid: a key signal molecule ameliorating plant stresses. Cereal Res Commun. 2022;50(4):617–26.

[21] Zhang X, Qi S, Liu S, Mu H, Jiang Y. Exogenous sodium nitroprusside alleviates drought stress in *Lagenaria siceraria*. Plants. 2024;13(14):1972. 39065499 10.3390/plants13141972 PMC11280828

[22] Siddiqui MH, Al-Whaibi MH, Basalah MO. Role of nitric oxide in tolerance of plants to abiotic stress. Protoplasma. 2011;248:447–55. 20827494 10.1007/s00709-010-0206-9

[23] Tan BC, Chin CF, Alderson P. Effects of sodium nitroprusside on shoot multiplication and regeneration of *Vanilla planifolia* Andrews. In Vitro Cell Dev Biol. 2013;49:626–30.

[24] Astier J, Gross I, Durner J. Nitric oxide production in plants: an update. J Exp Bot. 2018;69(14):3401–11. 29240949 10.1093/jxb/erx420

[25] Sundararajan S, Rajendran V, Sivakumar HP, Kumariah M, Ramalingam S. Growth modulation by nitric oxide donor sodium nitroprusside in in vitro plant tissue cultures–a review. Biologia. 2022;77(7):1699–711.

[26] Dempsey DMA, Vlot AC, Wildermuth MC, Klessig DF. Salicylic acid biosynthesis and metabolism. The Arabidopsis book/American Society of Plant Biologists. 2011;9:e0156. 10.1199/tab.0156 PMC3268552 22303280

[27] Miura K, Tada Y. Regulation of water, salinity, and cold stress responses by salicylic acid. Front Plant Sci. 2014;5:4. 24478784 10.3389/fpls.2014.00004 PMC3899523

[28] Vlot AC, Dempsey DMA, Klessig DF. Salicylic acid, a multifaceted hormone to combat disease. Annu Rev Phytopathol. 2009;47(1):177–206. 19400653 10.1146/annurev.phyto.050908.135202

[29] Raskin I, Skubatz H, Tang W, Meeuse BJ. Salicylic acid levels in thermogenic and non-thermogenic plants. Ann Bot. 1990;66(4):369–73.

[30] Raskin I. Role of salicylic acid in plants. Annu Rev Plant Biol. 1992;43(1):439–63.

[31] Popova L, Pancheva T, Uzunova A. Salicylic acid: properties, biosynthesis and physiological role. Bulg J Plant Physiol. 1997;23(1–2):85–93.

[32] HaraM, Furukawa J, Sato A, Mizoguchi T, Miura K. Abiotic Stress and Role of Salicylic Acid in Plants. In: Ahmad P, Prasad MNV, (Eds). Abiotic stress responses in plants: metabolism, productivity and sustainability. New York: Springer; 2012. p. 235–51. 10.1007/978-1-4614-0634-1_13.

[33] YusufM, Hayat S, Alyemeni MN, Fariduddin Q, Ahmad A. Salicylic acid: physiological roles in plants. In: Hayat S, Ahmad A, Alyemeni M, (eds) SALICYLIC ACID. Dordrecht: Springer; 2013. p. 15–30. 10.1007/978-94-007-6428-6_2.

[34] Bagautdinova ZZ, Omelyanchuk N, Tyapkin AV, Kovrizhnykh VV, Lavrekha VV, Zemlyanskaya EV. Salicylic acid in root growth and development. Int J Mol Sci. 2022;23(4):2228. 35216343 10.3390/ijms23042228 PMC8875895

[35] Mohammadi Cheraghabadi M, Roshanfekr H, Hasibi P, Meskarbashi M. Effect of foliar application of salicylic acid on some physiological traits of sugar beet in salt stress conditions. Iran J Field Crop Sci. 2015;46(4):591–604.

[36] Hepaksoy S. Bazı Kiraz anaçlarının mikroçoğaltımı üzerinde araştırmalar I. Gelişme ve çoğalma. Ege Üniversitesi Ziraat Fakültesi Dergisi. 2004;41(3):11–22.

[37] Ak BE, Kıyar PK, Hatipoglu IH, Dikmetaş B. Effects of different BA and IBA concentrations on proliferation and rooting of ‘GARNEM’ rootstock in vitro propagation. Int J Agric Environ Food Sci. 2021;5(4):470–6.

[38] Kara Z, Yazar K. In vitro poliploidy induction in some grape cultivars. Anadolu Tarım Bilimleri Dergisi. 2020;35(3):410–8.

[39] Kara. Z, Yazar K, Ekinci H, Doğan O, Özer A. The effects of ortho silicone applications on the acclimatization process of grapevine rootstocks. Selcuk J Agric Food Sci. 2022;36(2):233–7.

[40] Babu GA, Christas M, Kowsalya K, Ramesh E, Sohn M, S. I., Pandian S. Improved sterilization techniques for successful in vitro micropropagation. Commercial scale tissue culture for horticulture and plantation crops. Singapore: Springer Nature Singapore; 2022. pp. 1–21.

[41] Sarropoulou V, Maloupa E. Effect of the NO donor sodium Nitroprusside (SNP), the ethylene inhibitor Cobalt chloride (CoCl2) and the antioxidant vitamin E α-tocopherol on in vitro shoot proliferation of sideritis Raeseri Boiss. & Heldr. subsp. Raeseri. Plant Cell Tissue Organ Cult (PCTOC). 2017;128(3):619–29.

[42] Subiramani S, Sundararajan S, Sivakumar HP, et al. Sodium nitroprusside enhances callus induction and shoot regeneration in high value medicinal plant Canscora diffusa. Plant Cell Tiss Organ Cult. 2019;139:65–75. 10.1007/s11240-019-01663-x.

[43] Kazemi N, Jafarkhani Kermani M, Habashi AA. Sodium nitroprusside stimulates micropropagation and TDZ induces adventitious shoots regeneration in red flesh apple *Malus niedzwetzkyana* Koehne Dieck ex. J Hortic Res. 2019. 10.2478/johr-2019-0014.

[44] Jafari M, Shahsavar AR. Sodium nitroprusside: its beneficial role in drought stress tolerance of “Mexican lime” *Citrus aurantifolia *(Christ.) Swingle) under in vitro conditions. In Vitro Cellular & Developmental Biology-Plant. 2022;58(1):155–68.

[45] Eliwa GI, El Dengawy ERF, Gawish MS, Yamany MM. Comprehensive study on in vitro propagation of some imported peach rootstocks b. In vitro rooting and acclimatization. Sci Rep. 2025;15(1):17905. 40410261 10.1038/s41598-025-00848-z PMC12102309

[46] Lee S, Moon B, Kim S, Lee HW. Effects of rooting substrates and plant growth regulators on rooting performance, photosynthetic characteristics, and soil properties of Broussonetia× kazinoki Sieb. cuttings. Forests. 2025;16(11):1752.

[47] Ekinci H, Dikmetaş Doğan B, Şaşkın N, Aydınlık Y, Ak BE. Effects of healing agents on Aronia Melanocarpa Cv.‘Nero’under in vitro conditions against NaHCO_3_ induced alkaline stress. Appl Fruit Sci. 2025;67(2):1–9.

[48] Bukan M, Kereša S, Pejić I, Sudarić A, Lovrić A, Šarčević H. Variability of root and shoot traits under PEG-induced drought stress at an early vegetative growth stage of soybean. Agron J. 2024;14(6):1188.

[49] Erturk U, Yerlikaya C, Sivritepe N. In vitro phytoextraction capacity of blackberry for copper and zinc. Asian J Chem. 2007;19(3):2161.

[50] Sabra A, Daayf F, Renault S. Differential physiological and biochemical responses of three Echinacea species to salinity stress. Sci Hortic. 2012;135:23–31.

[51] Lutts S, Kinet JM, Bouharmont J. Effects of salt stress on growth, mineral nutrition and proline accumulation in relation to osmotic adjustment in rice (*Oryza sativa* L.) cultivars differing in salinity resistance. Plant Growth Regul. 1996;19:207–18.

[52] Sanchez FJ, De Andres EF, Tenorio JL, Ayerbe L. Growth of epicotyls, turgor maintenance and osmotic adjustment in pea plants (*Pisum sativum* L.) subjected to water stress. Field Crops Research. 2004;86(1):81–90.

[53] Demiral T, Türkan I. Comparative lipid peroxidation, antioxidant defense systems and proline content in roots of two rice cultivars differing in salt tolerance. Environ Exp Bot. 2005;53(3):247–57.

[54] Arnon DI. Copper enzymes in isolated chloroplasts. Polyphenoloxidase in beta vulgaris. Plant Physiol. 1949;24(1):1. 16654194 10.1104/pp.24.1.1 PMC437905

[55] Weisany W, Sohrabi Y, Heidari G, Siosemardeh A, Ghassemi-Golezani K. Changes in antioxidant enzymes activity and plant performance by salinity stress and zinc application in soybean (‘*Glycine max*’L). Plant Omics. 2012;5(2):60–7.

[56] Loreto F, Velikova V. Isoprene produced by leaves protects the photosynthetic apparatus against ozone damage, quenches ozone products, and reduces lipid peroxidation of cellular membranes. Plant Physiol. 2001;127(4):1781–7. 11743121 PMC133581

[57] Guo R, Shi L, Ding X, Hu Y, Tian S, Yan D, et al. Effects of saline and alkaline stress on germination, seedling growth, and ion balance in wheat. Agron J. 2010;102(4):1252–60.

[58] Shakirova FM. Role of hormonal system in the manifestation of growth promoting and antistress action of salicylic acid. In: Hayat S, Ahmad A, (eds) Salicylic acid: a plant hormone. Dordrecht: Springer; 2007. p. 69–89. 10.1007/1-4020-5184-0_4.

[59] Hayat Q, Hayat S, Irfan M, Ahmad A. Effect of exogenous salicylic acid under changing environment: a review. Environ Exp Bot. 2010;68(1):14–25.

[60] Sajid ZA, Aftab F. Role of salicylic acid in amelioration of salt tolerance in potato (*Solanum tuberosum* L.) under in vitro conditions. Pak J Bot. 2012;44:37–42.

[61] Yang C, Shi D, Wang D. Comparative effects of salt and alkali stresses on growth, osmotic adjustment and ionic balance of an alkali-resistant halophyte Suaeda glauca (Bge). Plant Growth Regul. 2008;56:179–90.

[62] Hasegawa PM. Sodium (Na+) homeostasis and salt tolerance of plants. Environ Exp Bot. 2013;92:19–31.

[63] Liu D, Cong RC, Dang HZ, Li QM, Liu DX, Yang QS. Comparative effects of salt and alkali stresses on plant physiology of Willow. Ecol Environ Sci. 2014;23:1531–5.

[64] Qin Y, Bai J, Wang Y, Liu J, Hu Y, Dong Z, et al. Comparative effects of salt and alkali stress on photosynthesis and root physiology of oat at anthesis. Arch Biol Sci. 2018;70(2):329–38.

[65] Yang S, Xu Y, Tang Z, Jin S, Yang S. The impact of alkaline stress on plant growth and its alkaline resistance mechanisms. Int J Mol Sci. 2024;25(24):13719. 39769481 10.3390/ijms252413719 PMC11677074

[66] Cristea TO, Iosob GA, Brezeanu C, Brezeanu PM, Bălăiță C, Calara M, Avasiloaiei DI. Assesment of various concentration of Salicylic acid in tissue culture in vitro systems for their effect on modulating abiotic stress tolerance mechanisms in pepper (Capsicum annuum L.) plants. Horticulture. 2025.Vol. LXIX, No. 1.

[67] Msimbira LA, Smith DL. The roles of plant growth promoting microbes in enhancing plant tolerance to acidity and alkalinity stresses. Frontiers in Sustainable Food Systems. 2020;4:106.

[68] Singh D, Singh CK, Singh YP, Singh V, Singh R, Tomar RSS, et al. Evaluation of cultivated and wild genotypes of *Lens* species under alkalinity stress and their molecular collocation using microsatellite markers. PLoS One. 2018;13(8):e0199933. 30102704 10.1371/journal.pone.0199933 PMC6089424

[69] Lamotte O, Courtois C, Dobrowolska G, Besson A, Pugin A, Wendehenne D. Mechanisms of nitric-oxide-induced increase of free cytosolic Ca2 + concentration in *Nicotiana Plumbaginifolia* cells. Free Radic Biol Med. 2006;40(8):1369–76. 16631527 10.1016/j.freeradbiomed.2005.12.006

[70] Chavoushi M, Najafi F, Salimi A, Angaji SA. Effect of salicylic acid and sodium nitroprusside on growth parameters, photosynthetic pigments and secondary metabolites of safflower under drought stress. Sci Hortic. 2020;259:108823.

[71] Arun M, Naing AH, Jeon SM, Ai TN, Aye T, Kim CK. Sodium nitroprusside stimulates growth and shoot regeneration in chrysanthemum. Hortic Environ Biotechnol. 2017;58:78–84.

[72] Scherer GF, Holk A. NO donors mimic and NO inhibitors inhibit cytokinin action in betalaine accumulation in *Amaranthus caudatus*. Plant Growth Regul. 2000;32:345–50.

[73] Ghosh P, Roychoudhury A. Molecular basis of salicylic acid–phytohormone crosstalk in regulating stress tolerance in plants. Braz J Bot. 2024;47(3):735–50.

[74] Koo YM, Heo AY, Choi HW. Salicylic acid as a safe plant protector and growth regulator. Plant Pathol J. 2020;36(1):1. 32089657 10.5423/PPJ.RW.12.2019.0295 PMC7012573

[75] Phong Lam V, Loi DN, Shin J, Mi LK, Park J. Optimization of salicylic acid concentrations for increasing antioxidant enzymes and bioactive compounds of *Agastache rugosa* in a plant factory. PLoS One. 2024;19(7):e0306340. 39052558 10.1371/journal.pone.0306340 PMC11271957

[76] Moharramnejad S, Azam AT, Panahandeh J, Dehghanian Z, Ashraf M. Effect of methyl jasmonate and salicylic acid on in vitro growth, stevioside production, and oxidative defense system in *Stevia rebaudiana*. Sugar Tech. 2019;21(6):1031–8.

[77] Cantal D. Effect of different rootstocks on fruit quality and plant nutritient content of almond cvs. Ferragnes and Ferraduel (Doctoral dissertation, M. Sc. Thesis, Ege University Graduate School of Applied and Natural Science). 2022. p. 56.

[78] Xu LL, Fan ZY, Dong YJ, Kong J, Bai XY. Effects of exogenous Salicylic acid and nitric oxide on physiological characteristics of two peanut cultivars under cadmium stress. Biol Plant. 2015;59(1):171–82.

[79] Yang H, Fang R, Luo L, Yang W, Huang Q, Yang C, et al. Uncovering the mechanisms of salicylic acid-mediated abiotic stress tolerance in horticultural crops. Front Plant Sci. 2023;14:1226041. 37701800 10.3389/fpls.2023.1226041 PMC10494719

[80] Antonić D, Milošević S, Cingel A, Lojić M, Trifunović-Momčilov M, Petrić M, et al. Effects of exogenous salicylic acid on *Impatiens walleriana* L. grown in vitro under polyethylene glycol-imposed drought. S Afr J Bot. 2016;105:226–33.

[81] Ötvös K, Pasternak TP, Miskolczi P, Domoki M, Dorjgotov D, Sz˝ cs A, et al. Nitric oxide is required for, and promotes auxin-mediated activation of, cell division and embryogenic cell formation but does not influence cell cycle progression in alfalfa cell cultures. Plant J. 2005;43(6):849–60. 16146524 10.1111/j.1365-313X.2005.02494.x

[82] Xu J, Yin H, Wang W, Mi Q, Liu X. Effects of sodium Nitroprusside on callus induction and shoot regeneration in micropropagated *Dioscorea opposita*. Plant Growth Regul. 2009;59:279–85.

[83] Sarropoulou V, Dimassi-Theriou K, Therios I. Ιn vitro plant regeneration from leaf explants of the Cherry rootstocks CAB-6P, Gisela 6, and MxM 14 using sodium Nitroprusside. In Vitro Cell Dev Biol-Plant. 2014;50:226–34.

[84] Hamilton EW III, Heckathorn SA. Mitochondrial adaptations to NaCl. Complex I is protected by anti-oxidants and small heat shock proteins, whereas complex II is protected by proline and betaine. Plant Physiol. 2001;126(3):1266–74. 11457977 10.1104/pp.126.3.1266 PMC116483

[85] Poór P. Effects of Salicylic acid on the metabolism of mitochondrial reactive oxygen species in plants. Biomolecules. 2020;10(2):341. 32098073 10.3390/biom10020341 PMC7072379

[86] Lombardo MC, Graziano M, Polacco JC, Lamattina L. Nitric oxide functions as a positive regulator of root hair development. Plant Signal Behav. 2006;1(1):28–33. 19521473 10.4161/psb.1.1.2398 PMC2633697

[87] Sadat-Hosseini M, Soleimani A. Callus induction, shoot and root regeneration in *Hyssopus officinalis* using Sodium Nitroprusside and Plant Growth Regulators. Journal of Medicinal plants and By-Products. 2024;13(4):932–9.

[88] Sakhanokho HF, Kelley RY. Influence of Salicylic acid on in vitro propagation and salt tolerance in hibiscus acetosella and hibiscus Moscheutos (cv ‘Luna Red’). Afr J Biotechnol. 2009;8(8):1474–81.

[89] Lin J, Li X, Zhang Z, Yu X, Gao Z, Wang Y, Mu C. Salinity-alkalinity tolerance in wheat: Seed germination, early seedling growth, ion relations and solute accumulation. Afr J Agric Res. 2012;7(3):467–74.

[90] Ahmad P, Ozturk M, Sharma S, Gucel S. Effect of sodium carbonate-induced salinity–alkalinity on some key osmoprotectants, protein profile, antioxidant enzymes, and lipid peroxidation in two mulberry (*Morus alba* L.) cultivars. J Plant Interact. 2014;9(1):460–7.

[91] Cartmill AD, Valdez-Aguilar LA, Bryan DL, Alarcón A. Arbuscular mycorrhizal fungi enhance tolerance of *Vinca* to high alkalinity in irrigation water. Sci Hort. 2008;115(3):275–84.

[92] Pérez-Martín L, Busoms S, Tolrà R, Poschenrieder C. Transcriptomics reveals fast changes in salicylate and jasmonate signaling pathways in shoots of carbonate-tolerant *Arabidopsis thaliana* under bicarbonate exposure. Int J Mol Sci. 2021;22(3):1226. 33513755 10.3390/ijms22031226 PMC7865540

[93] Sagervanshi A, Naeem A, Kaiser H, Pitann B, Mühling KH. Early growth reduction in *Vicia faba* L. under alkali salt stress is mainly caused by excess bicarbonate and related to citrate and malate over accumulation. Environ Exp Bot. 2021;192:104636.

[94] Dhankher OP, Foyer CH. Climate resilient crops for improving global food security and safety. Plant Cell Environ. 2018;41(5):877–84. 10.1111/pce.13207 29663504

[95] Ullah F, Saqib S, Khan W, Ayaz A, Batool A, Wang WY, et al. The multifaceted role of sodium nitroprusside in plants: crosstalk with phytohormones under normal and stressful conditions. Plant Growth Regul. 2024;103(3):453–70.

[96] Ghadakchiasl A, Mozafari AA, Ghaderi N. Mitigation by sodium nitroprusside of the effects of salinity on the morpho-physiological and biochemical characteristics of *Rubus idaeus* under in vitro conditions. Physiol Mol Biol Plants. 2017;23:73–83. 28250585 10.1007/s12298-016-0396-5 PMC5313400

[97] Radojičić A, Li X, Zhang Y. Salicylic acid: a double-edged sword for programed cell death in plants. Front Plant Sci. 2018;9:1133. 30131819 10.3389/fpls.2018.01133 PMC6090181

[98] Ahmad P, Abdel Latef AA, Hashem A, Abd_Allah EF, Gucel S, Tran LSP. Nitric oxide mitigates salt stress by regulating levels of osmolytes and antioxidant enzymes in chickpea. Front Plant Sci. 2016;7:347. 27066020 10.3389/fpls.2016.00347 PMC4814448

[99] Kuşvuran Ş. Kavunlarda kuraklık ve tuzluluğa toleransın fizyolojik mekanizmaları arasındaki bağlantılar, doktora Tezi, Çukurova Üniversitesi. Adana: Fen Bilimleri Enstitüsü; 2010. p. 356s.

[100] Mittler R. Oxidative stress, antioxidants and stress tolerance. Trends Plant Sci. 2002;7:405–10. 12234732 10.1016/s1360-1385(02)02312-9

[101] Gill SS, Tuteja N. Reactive oxygen species and antioxidant machinery in abiotic stress tolerance in crop plants. Plant Physiol Biochem. 2010;48(12):909–30. 20870416 10.1016/j.plaphy.2010.08.016

[102] Wani KI, Naeem M, Castroverde CDM, Kalaji HM, Albaqami M, Aftab T. Molecular mechanisms of nitric oxide (NO) signaling and reactive oxygen species (ROS) homeostasis during abiotic stresses in plants. Int J Mol Sci. 2021;22(17):9656. 34502565 10.3390/ijms22179656 PMC8432174

[103] Kumar S, Ahanger MA, Alshaya H, Jan BL, Yerramilli V. Salicylic acid mitigates salt induced toxicity through the modifications of biochemical attributes and some key antioxidants in *capsicum annuum*. Saudi J Biol Sci. 2022;29(3):1337–47. 35280588 10.1016/j.sjbs.2022.01.028 PMC8913376

[104] Çırak C, Esendal E. Soyada Kuraklik Stresi. Anadolu Tarım Bilimleri Dergisi. 2006;21(2):231–7.

[105] Lau SE, Hamdan MF, Pua TL, Saidi NB, Tan BC. Plant nitric oxide signaling under drought stress. Plants. 2021;10(2):360. 33668545 10.3390/plants10020360 PMC7917642

[106] Zhang Y, Xu J, Li R, Ge Y, Li Y, Li R. Plants’ response to abiotic stress: mechanisms and strategies. Int J Mol Sci. 2023;24(13):10915. 37446089 10.3390/ijms241310915 PMC10341657

[107] Pandey A, Khan MK, Hamurcu M, Athar T, Yerlikaya BA, Yerlikaya S, et al. Role of exogenous nitric oxide in protecting plants against abiotic stresses. Agronomy. 2023;13(5):1201.

[108] Khokon MAR, Okuma EIJI, Hossain MA, Munemasa S, Uraji M, Nakamura Y, et al. Involvement of extracellular oxidative burst in salicylic acid-induced stomatal closure in Arabidopsis. Plant, cell & environment. 2011;34(3):434–43. 10.1111/j.1365-3040.2010.02253.x 21062318

[109] Loutfy N, El-Tayeb MA, Hassanen AM, Moustafa MF, Sakuma Y, Inouhe M. Changes in the water status and osmotic solute contents in response to drought and Salicylic acid treatments in four different cultivars of wheat (*Triticum aestivum*). J Plant Res. 2012;125:173–84. 21445718 10.1007/s10265-011-0419-9

[110] Simkin AJ, López-Calcagno PE, Raines CA. Feeding the world: improving photosynthetic efficiency for sustainable crop production. J Exp Bot. 2019;70(4):1119–40. 30772919 10.1093/jxb/ery445 PMC6395887

[111] Hussain S, Ulhassan Z, Brestic M, Zivcak M, Zhou W, Allakhverdiev SI, et al. Photosynthesis research under climate change. Photosynth Res. 2021;150:5–19. 34235625 10.1007/s11120-021-00861-z

[112] Chen W, Feng C, Guo W, Shi D, Yang C. Comparative effects of osmotic-, salt-and alkali stress on growth, photosynthesis, and osmotic adjustment of cotton plants. Photosynthetica. 2011;49:417–25.

[113] Yang JY, Zheng W, Tian Y, Wu Y, Zhou DW. Effects of various mixed salt-alkaline stresses on growth, photosynthesis, and photosynthetic pigment concentrations of *Medicago ruthenica* seedlings. Photosynthetica. 2011;49:275–84.

[114] Liu D, Ma Y, Rui M, Lv X, Chen R, Chen X, et al. Is high pH the key factor of alkali stress on plant growth and physiology? A case study with wheat (*Triticum aestivum* L.) seedlings. Agronomy. 2022;12(8):1820.

[115] Mohammadi-Cheraghabadi M, Modarres‐Sanavy SAM, Sefidkon F, Mokhtassi‐Bidgoli A, Hazrati S. Effects of water‐deficit stress and Putrescine on performances, photosynthetic gas exchange, and chlorophyll fluorescence parameters of *Salvia officinalis* in two cutting times. Food Sci Nutr. 2022;10(5):1431. 35592300 10.1002/fsn3.2741 PMC9094464

[116] Rouphael Y, Cardarelli M, Di Mattia E, Tullio M, Rea E, Colla G. Enhancement of alkalinity tolerance in two cucumber genotypes inoculated with an arbuscular mycorrhizal biofertilizer containing *Glomus intraradices*. Biol Fertil Soils. 2010;46:499–509.

[117] Liu L, Saneoka H. Effects of NaHCO_3_ acclimation on Rye (*Secale cereale*) growth under sodic-alkaline stress. Plants. 2019;8(9):314. 31480305 10.3390/plants8090314 PMC6784086

[118] Kausar F, Shahbaz M, Ashraf M. Protective role of foliar-applied nitric oxide in *Triticum aestivum* under saline stress. Turk J Bot. 2013;37(6):1155–65.

[119] Ahmad P, Ahanger MA, Alyemeni MN, Wijaya L, Alam P. Exogenous application of nitric oxide modulates osmolyte metabolism, antioxidants, enzymes of ascorbate-glutathione cycle and promotes growth under cadmium stress in tomato. Protoplasma. 2018;255:79–93. 28643085 10.1007/s00709-017-1132-x

[120] Janda T, Gondor OK, Yordanova R, Szalai G, Pál M. Salicylic acid and photosynthesis: signalling and effects. Acta Physiol Plant. 2014;36:2537–46.

[121] Miller GAD, Suzuki N, Ciftci-Yilmaz SULTAN, Mittler RON. Reactive oxygen species homeostasis and signalling during drought and salinity stresses. Plant Cell Environ. 2010;33(4):453–67. 19712065 10.1111/j.1365-3040.2009.02041.x

[122] Sharma P, Jha AB, Dubey RS, Pessarakli M. Reactive oxygen species, oxidative damage, and antioxidative defense mechanism in plants under stressful conditions. J Bot. 2012;2012(1):217037.

[123] Saxena I, Srikanth S, Chen Z. Cross talk between H_2_O_2_ and interacting signal molecules under plant stress response. Front Plant Sci. 2016;7:570. 27200043 10.3389/fpls.2016.00570 PMC4848386

[124] Choudhury FK, Rivero RM, Blumwald E, Mittler R. Reactive oxygen species, abiotic stress and stress combination. Plant J. 2017;90(5):856–67. 27801967 10.1111/tpj.13299

[125] Liu XL, Zhang H, Jin YY, Wang MM, Yang HY, Ma HY, et al. Abscisic acid primes rice seedlings for enhanced tolerance to alkaline stress by upregulating antioxidant defense and stress tolerance-related genes. Plant Soil. 2019;438:39–55.

[126] Mohammadi-Cheraghabadi M, Modarres-Sanavy SAM, Sefidkon F, Rashidi-Monfared S, Mokhtassi-Bidgoli A. Improving water deficit tolerance of *Salvia officinalis* L. using putrescine. Sci Rep. 2021;11(1):21997. 34753954 10.1038/s41598-021-00656-1 PMC8578639

[127] SarıoğluA. Bitki Büyümesini ve Stres Toleransını Arttırmak: Abiyotik Stres Altında Biber ve Soya Bitkilerinde Melatonin, Salisilik Asit ve Bakteri Aşılamasının Etkilerinin İncelenmesi. Harran University, Institute of Science, Department of Soil Science and Plant Nutrition, PhD Thesis. 2023. p. 187. http://hdl.handle.net/11513/3598.

[128] Kotapati KV, Palaka BK, Ampasala DR. Alleviation of nickel toxicity in finger millet (*Eleusine coracana* L.) germinating seedlings by exogenous application of salicylic acid and nitric oxide. Crop J. 2017;5(3):240–50.

[129] Ghassemi-Golezani K, Farhadi N, Nikpour-Rashidabad N. Responses of in vitro-cultured *Allium hirtifolium* to exogenous sodium nitroprusside under PEG-imposed drought stress. Plant Cell, Tissue and Organ Culture (PCTOC). 2018;133(2):237–48.

[130] Morales M, Munné-Bosch S. Malondialdehyde: facts and artifacts. Plant Physiol. 2019;180(3):1246–50. 31253746 10.1104/pp.19.00405 PMC6752910

[131] Zhang Y, Luan Q, Jiang J, Li Y. Prediction and utilization of malondialdehyde in exotic pine under drought stress using near-infrared spectroscopy. Front Plant Sci. 2021;12:735275. 34733301 10.3389/fpls.2021.735275 PMC8558207

[132] Zhang L, Qian Q, Song S. Combating alkaline stress with alkaline tolerance 1, which encodes a conserved Gγ protein in multiple crops. Plant Communications. 2023. 10.1016/j.xplc.2023.100603. 37085992 10.1016/j.xplc.2023.100603 PMC10203448

[133] Sanglyne MW, Das MC. Unraveling the impact of sodium nitroprusside on morphogenesis, selected phytochemical profiling, and antioxidant activities of in vitro–raised plantlets of citrus *indica* Yu. Tanaka. In Vitro Cell Dev Biol-Plant. 2024;60(1):98–111.

[134] Horváth E, Szalai G, Janda T. Induction of abiotic stress tolerance by salicylic acid signaling. J Plant Growth Regul. 2007;26(3):290–300.

[135] Khan MIR, Fatma M, Per TS, Anjum NA, Khan NA. Salicylic acid-induced abiotic stress tolerance and underlying mechanisms in plants. Front Plant Sci. 2015;6:462. 26175738 10.3389/fpls.2015.00462 PMC4485163

[136] Li R, Shi F, Fukuda K. Interactive effects of various salt and alkali stresses on growth, organic solutes, and cation accumulation in a halophyte spartina *alterniflora* (Poaceae). Environ Exp Bot. 2010;68(1):66–74. 10.1016/j.envexpbot.2009.10.004.

[137] Hasegawa PM, Bressan RA, Zhu JK, Bohnert HJ. Plant cellular and molecular responses to high salinity. Annu Rev Plant Biol. 2000;51(1):463–99. 10.1146/annurev.arplant.51.1.463. 10.1146/annurev.arplant.51.1.463 15012199

[138] Athar HUR, Khan A, Ashraf M. Inducing salt tolerance in wheat by exogenously applied ascorbic acid through different modes. J Plant Nutrition. 2009;32(11):1799–1817. 10.1080/01904160903242334.

[139] Jiménez S, Dridi J, Gutiérrez D, Moret D, Irigoyen JJ, Moreno MA, Gogorcena Y. Physiological, biochemical and molecular responses in four Prunus rootstocks submitted to drought stress. Tree Physiol. 2013;33(10):1061–75. 24162335 10.1093/treephys/tpt074

[140] He Z, Webster S, He SY. Growth–defense trade-offs in plants. Curr Biol. 2022;32(12):R634–9. 35728544 10.1016/j.cub.2022.04.070

[141] Gerloff-Elias ANTJE, Spijkerman ELLY, Pröschold T. Effect of external pH on the growth, photosynthesis and photosynthetic electron transport of *chlamydomonas acidophila* Negoro, isolated from an extremely acidic lake (pH 2.6). Plant Cell Environ. 2005;28(10):1218–29.

[142] Malekzadeh Shamsabad MR, Roosta HR, Esmaeilizadeh M. Responses of seven strawberry cultivars to alkalinity stress under soilless culture system. J Plant Nutr. 2021;44(2):166–80. 10.1080/01904167.2020.1822401.

[143] Hasanuzzaman M, Oku H, Nahar K, Bhuyan MB, Mahmud JA, Baluska F, Fujita M. Nitric oxide-induced salt stress tolerance in plants: ROS metabolism, signaling, and molecular interactions. Plant Biotechnol Rep. 2018;12(2):77–92. 10.1007/s11816-018-0480-0.

