# Supplementary material for: Effects of sodium nitroprusside and salicylic acid applications on morphological, physiological and biochemical properties of Garnem (Prunus dulcis × Prunus persica) rootstock against alkaline stress under in vitro conditions
Source: BMC Plant Biol. 2026 Feb 18;26:553. doi: 10.1186/s12870-026-08300-8 (PMC13019762; doi:10.1186/s12870-026-08300-8)
Supplement: Supplementary file 1 — Supplementary Material 1 [file 12870_2026_8300_MOESM1_ESM.docx]

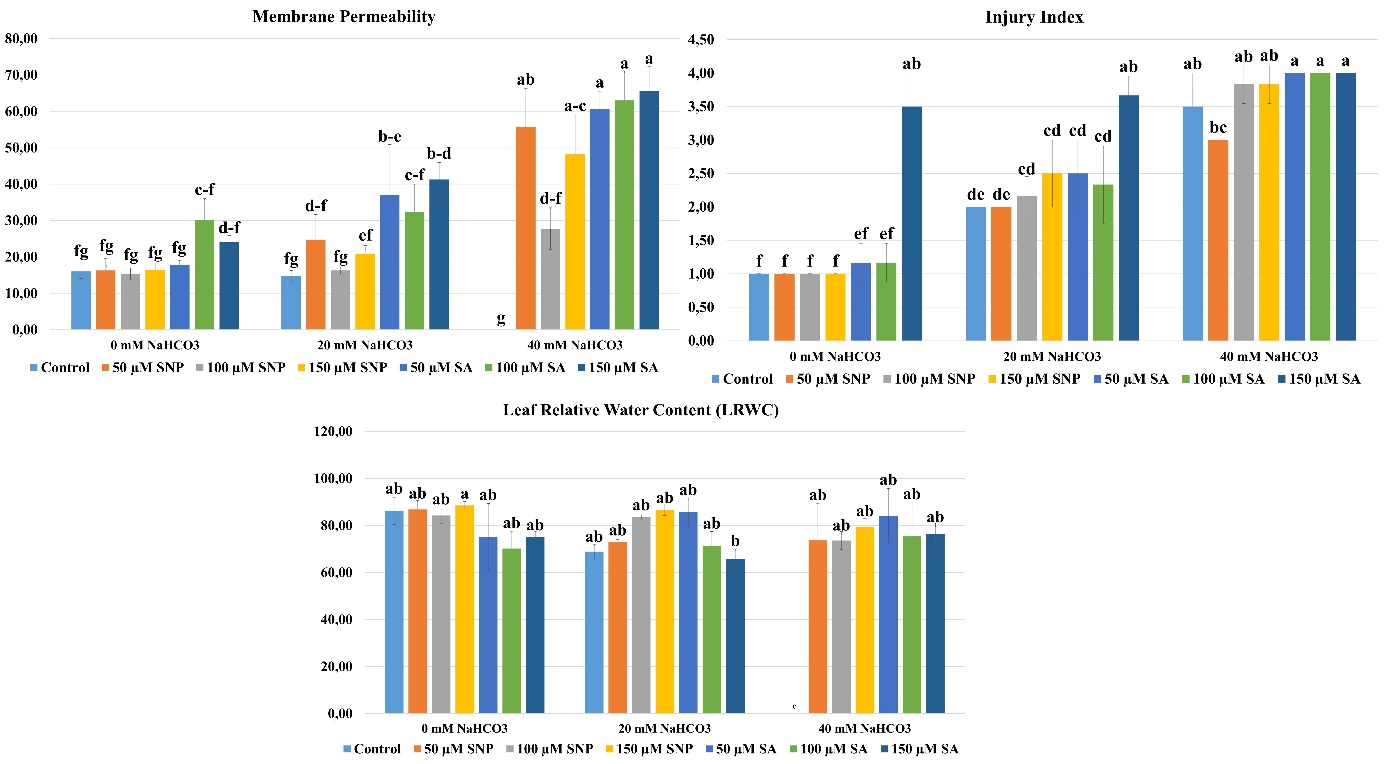


**Figure 5.** Effect of SNP and SA applications on injury index, membrane permeability and LRWC parameters under NaHCO_3_-induced alkaline stress conditions *in vitro* (LRWC: Leaf Relative Water Content) (p≤0.05).
