# Supplementary material for: Effects of sodium nitroprusside and salicylic acid applications on morphological, physiological and biochemical properties of Garnem (Prunus dulcis × Prunus persica) rootstock against alkaline stress under in vitro conditions
Source: BMC Plant Biol. 2026 Feb 18;26:553. doi: 10.1186/s12870-026-08300-8 (PMC13019762; doi:10.1186/s12870-026-08300-8)
Supplement: Supplementary file 2 — Supplementary Material 2 [file 12870_2026_8300_MOESM2_ESM.pdf]

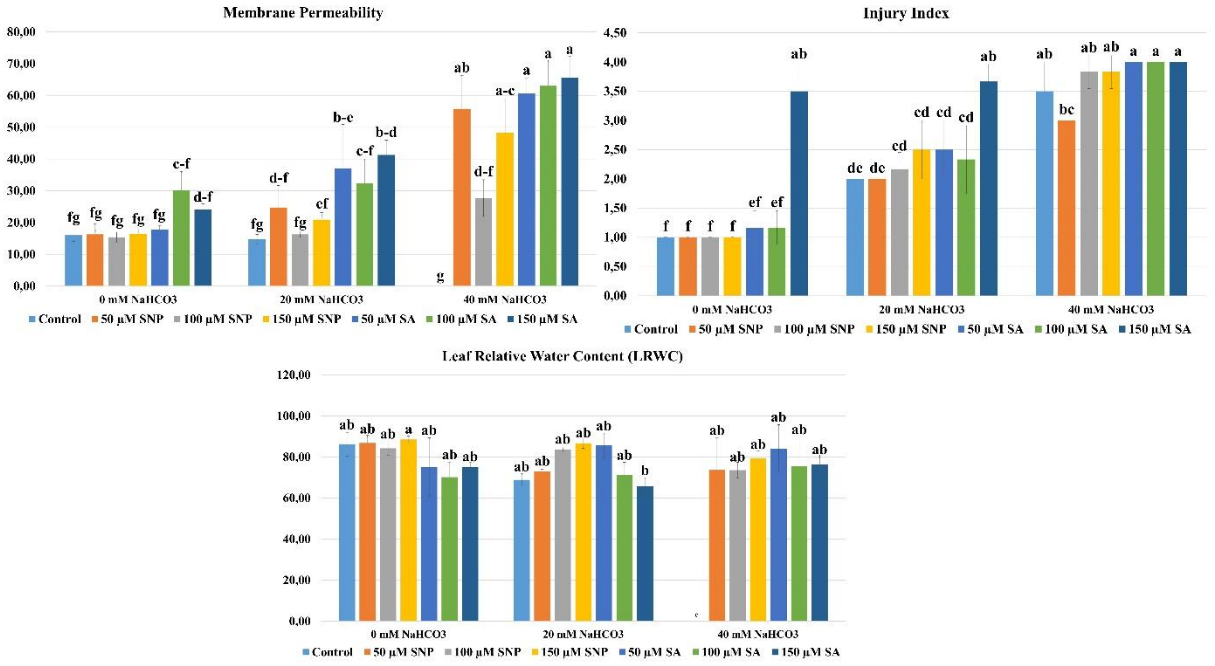

Figure 5. Effect of SNP and SA applications on injury index, membrane permeability and LRWC parameters under $NaHCO_3$-induced alkaline stress conditions *in vitro* (LRWC: Leaf Relative Water Content) ($p \leq 0.05$).

* The letter groupings used in the statistical analysis indicate differences among the $NaHCO_3$ × SNP/SA interactions. According to Tukey's multiple comparison test, mean values denoted by different letters differ significantly at the 5% significance level.
