# Supplementary figures and images for: Effects of sodium nitroprusside and salicylic acid applications on morphological, physiological and biochemical properties of Garnem (Prunus dulcis × Prunus persica) rootstock against alkaline stress under in vitro conditions
Source: BMC Plant Biol. 2026 Feb 18;26:553. doi: 10.1186/s12870-026-08300-8 (PMC13019762; doi:10.1186/s12870-026-08300-8)

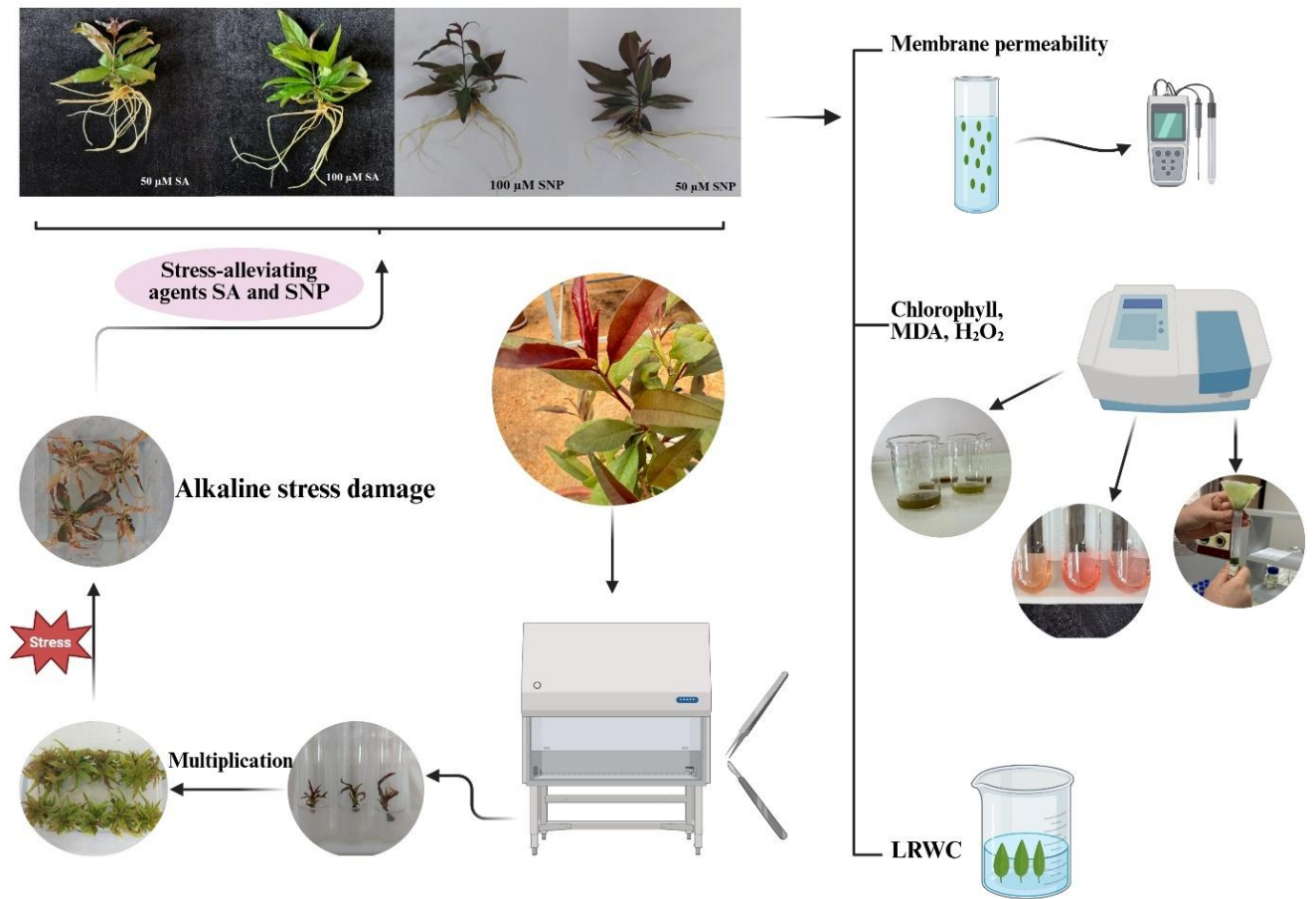

Supplement: Supplementary file 3 — Supplementary Material 3 [file 12870_2026_8300_MOESM3_ESM.pdf]

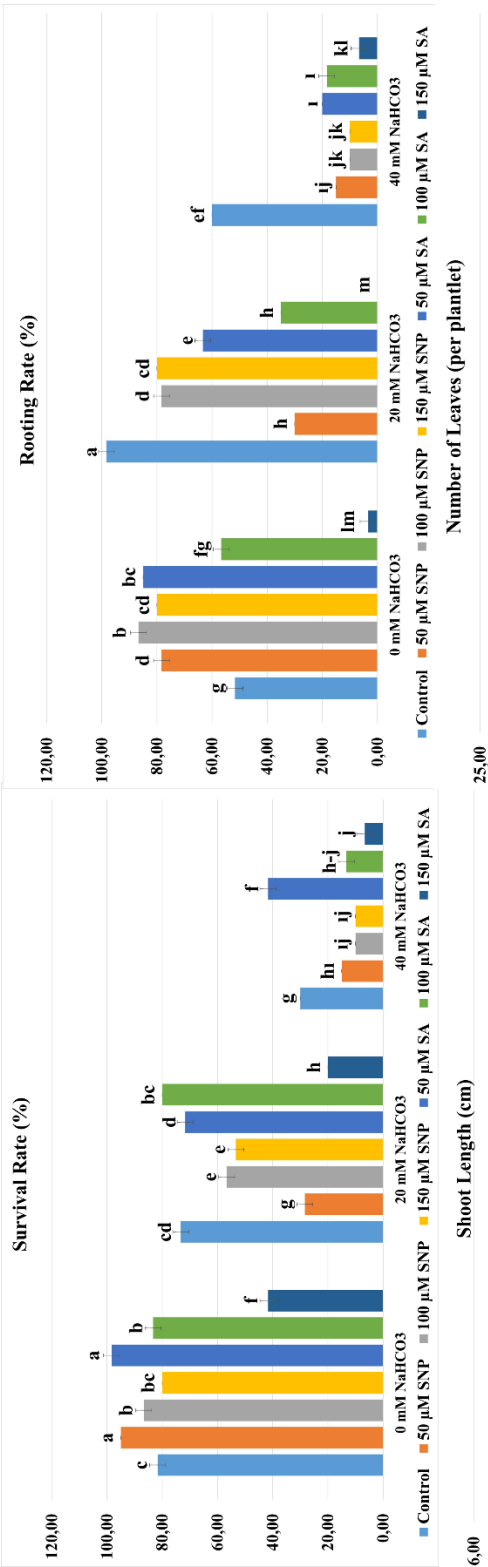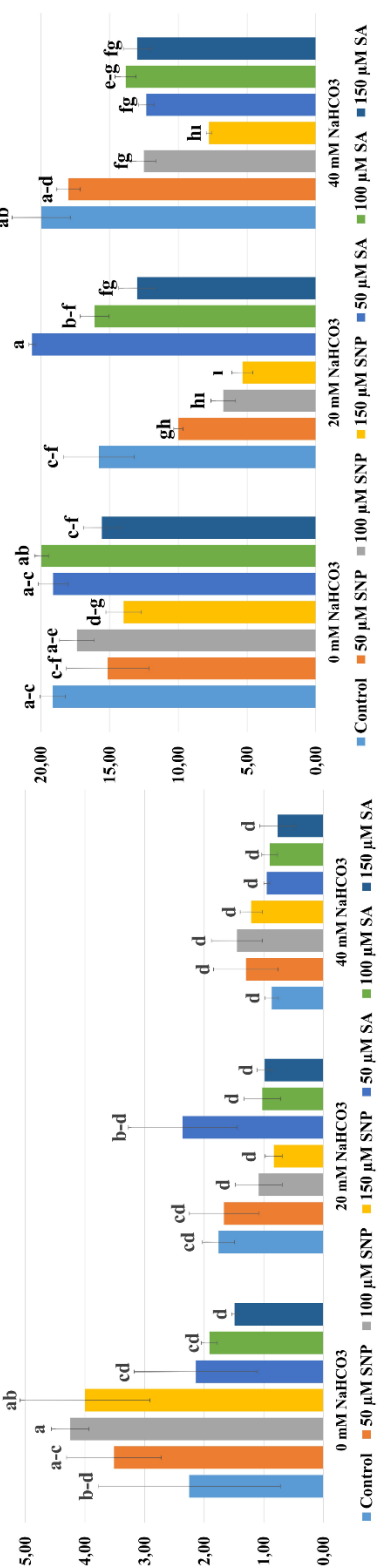

Supplement: Supplementary file 4 — Supplementary Material 4 [file 12870_2026_8300_MOESM4_ESM.pdf]

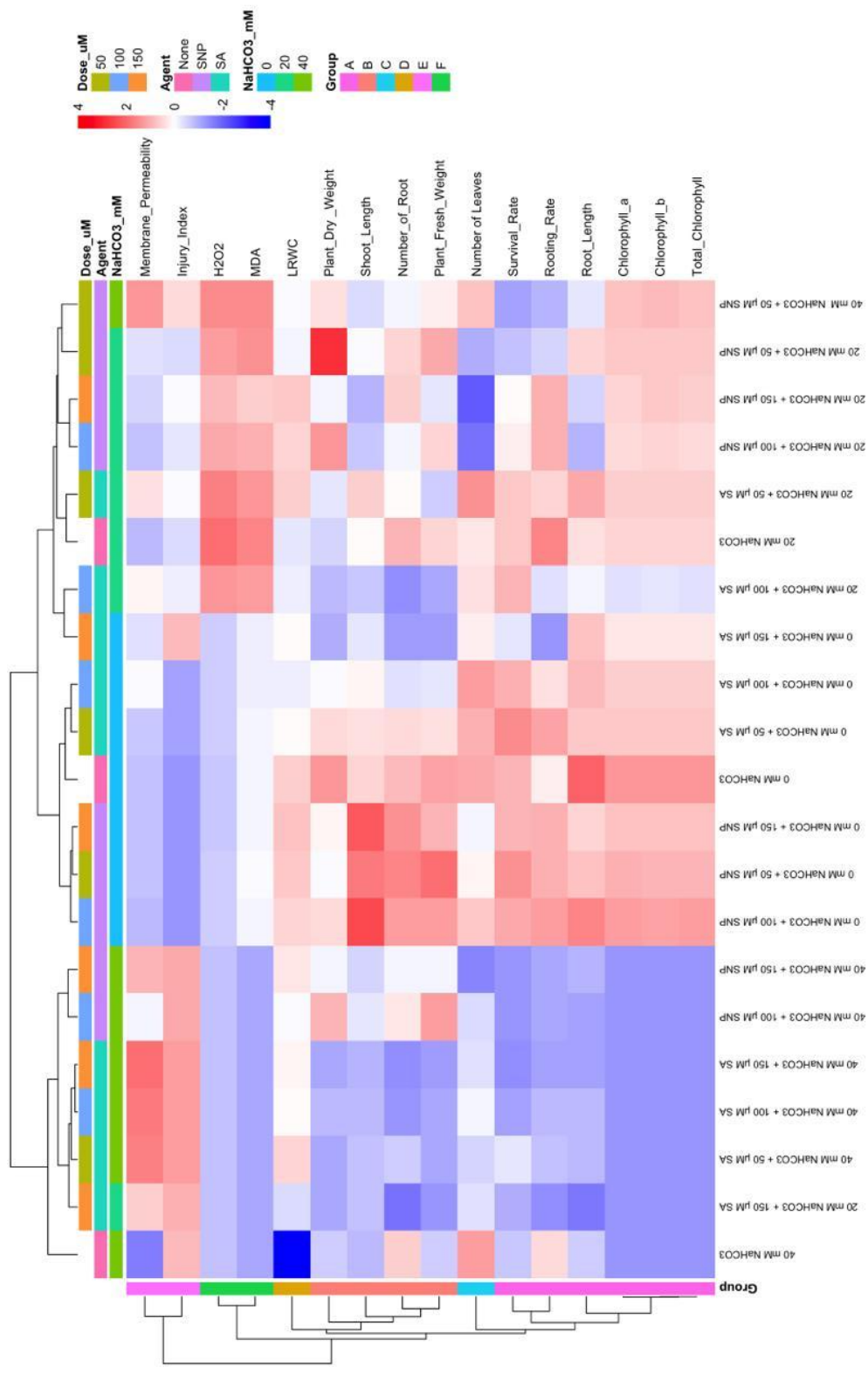

Supplement: Supplementary file 5 — Supplementary Material 5 [file 12870_2026_8300_MOESM5_ESM.pdf]

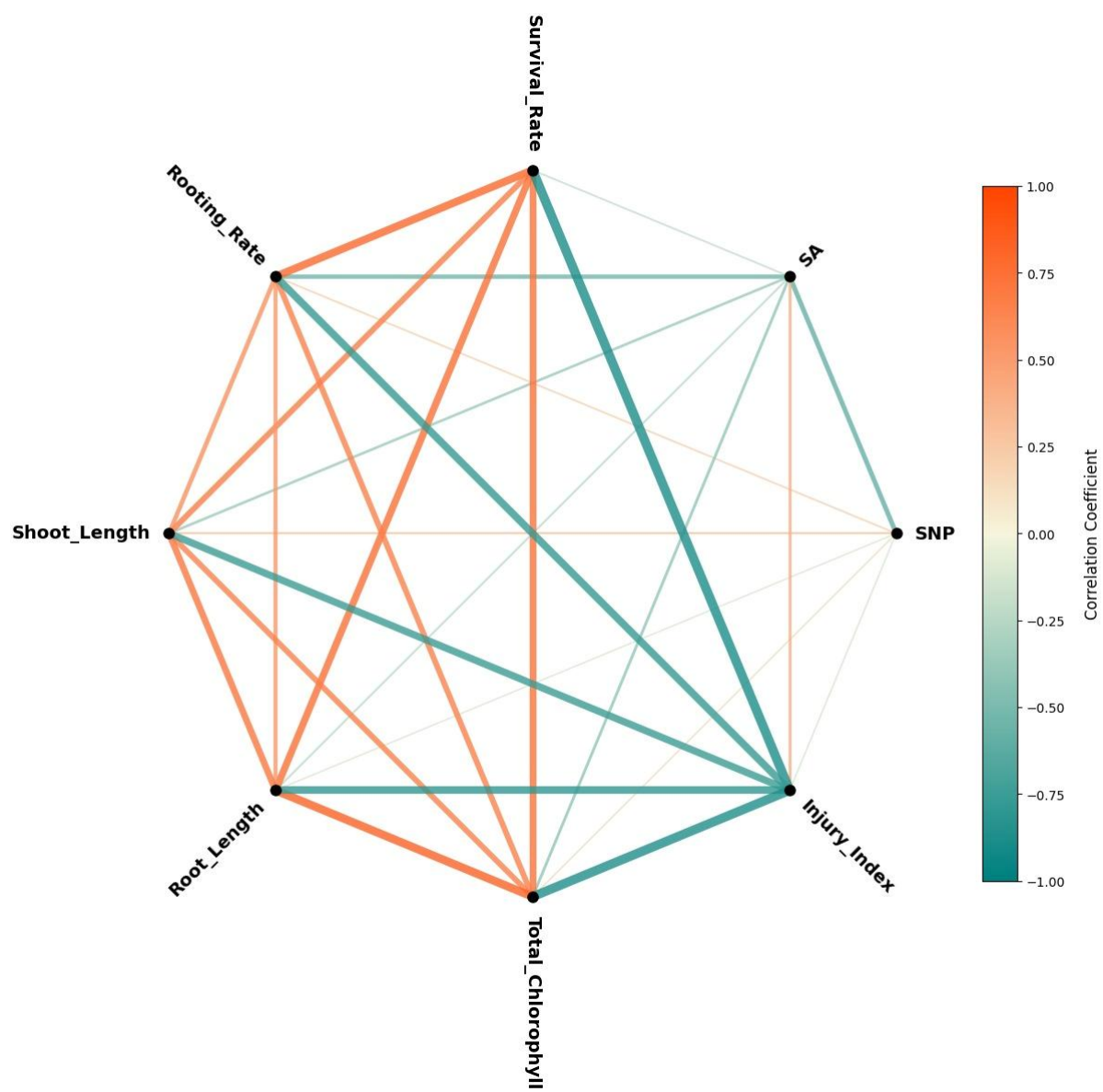

## Hive Plot of Parameter Correlations

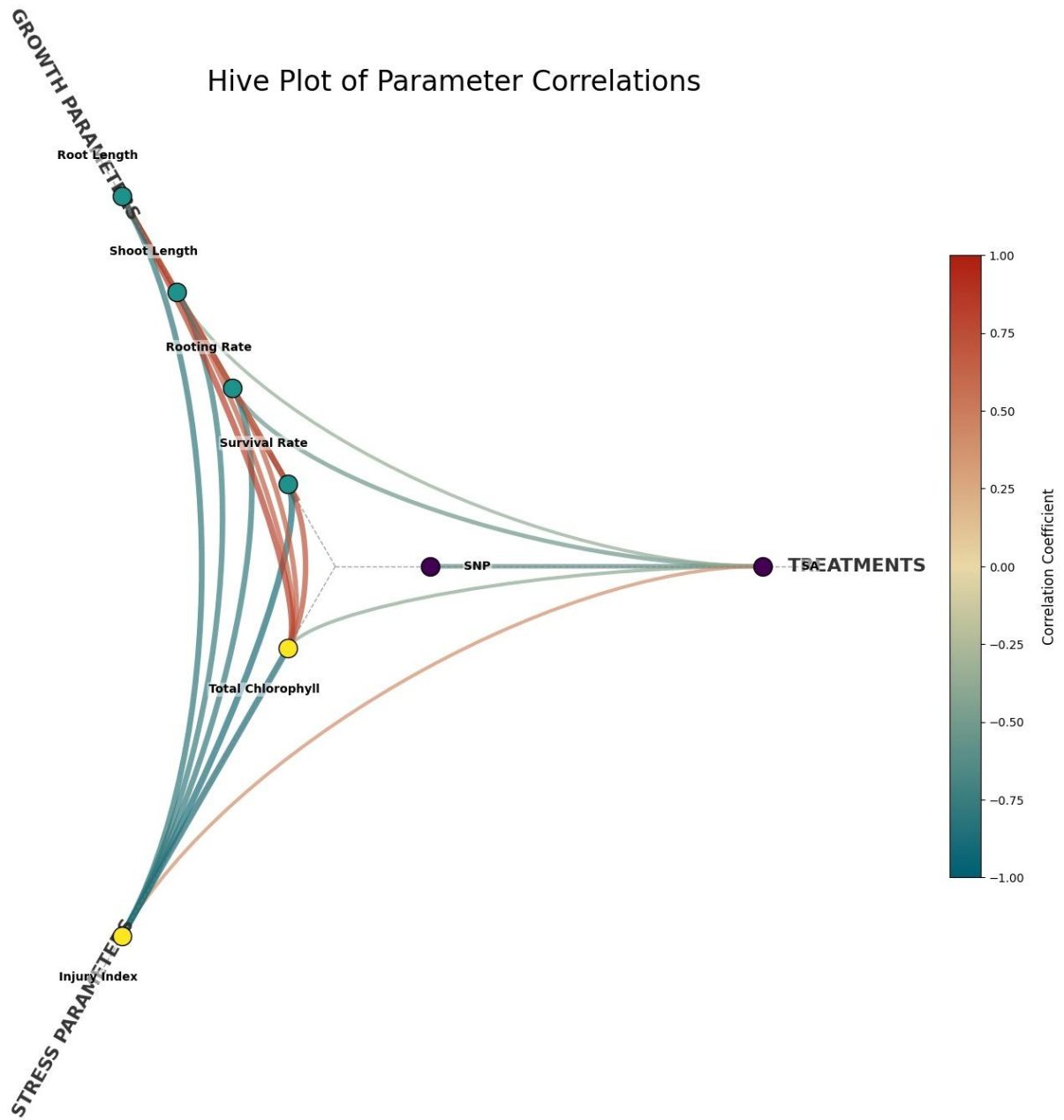

Supplement: Supplementary file 6 — Supplementary Material 6 [file 12870_2026_8300_MOESM6_ESM.pdf]
